# Continuous time boolean modeling for biological signaling: application of Gillespie algorithm

**DOI:** 10.1186/1752-0509-6-116

**Published:** 2012-08-29

**Authors:** Gautier Stoll, Eric Viara, Emmanuel Barillot, Laurence Calzone

**Affiliations:** 1 Institut Curie, 26 rue d’Ulm, Paris, F-75248 France; 2 INSERM, Paris, U900, F-75248 France; 3 Mines ParisTech, Fontainebleau, F-77300 France; 4 Sysra, Yerres, F-91330 France

**Keywords:** Boolean modeling, Continuous time, Markov process, Gillespie algorithm

## Abstract

Mathematical modeling is used as a Systems Biology tool to answer biological questions, and more precisely, to validate a network that describes biological observations and predict the effect of perturbations. This article presents an algorithm for modeling biological networks in a discrete framework with continuous time.

**Background:**

There exist two major types of mathematical modeling approaches: (1) quantitative modeling, representing various chemical species concentrations by real numbers, mainly based on differential equations and chemical kinetics formalism; (2) and qualitative modeling, representing chemical species concentrations or activities by a finite set of discrete values. Both approaches answer particular (and often different) biological questions. Qualitative modeling approach permits a simple and less detailed description of the biological systems, efficiently describes stable state identification but remains inconvenient in describing the transient kinetics leading to these states. In this context, time is represented by discrete steps. Quantitative modeling, on the other hand, can describe more accurately the dynamical behavior of biological processes as it follows the evolution of concentration or activities of chemical species as a function of time, but requires an important amount of information on the parameters difficult to find in the literature.

**Results:**

Here, we propose a modeling framework based on a qualitative approach that is intrinsically continuous in time. The algorithm presented in this article fills the gap between qualitative and quantitative modeling. It is based on continuous time Markov process applied on a Boolean state space. In order to describe the temporal evolution of the biological process we wish to model, we explicitly specify the transition rates for each node. For that purpose, we built a language that can be seen as a generalization of Boolean equations. Mathematically, this approach can be translated in a set of ordinary differential equations on probability distributions. We developed a C++ software, MaBoSS, that is able to simulate such a system by applying Kinetic Monte-Carlo (or Gillespie algorithm) on the Boolean state space. This software, parallelized and optimized, computes the temporal evolution of probability distributions and estimates stationary distributions.

**Conclusions:**

Applications of the Boolean Kinetic Monte-Carlo are demonstrated for three qualitative models: a toy model, a published model of p53/Mdm2 interaction and a published model of the mammalian cell cycle. Our approach allows to describe kinetic phenomena which were difficult to handle in the original models. In particular, transient effects are represented by time dependent probability distributions, interpretable in terms of cell populations.

## Background

Mathematical models of signaling pathways are tools that answer biological questions. The most commonly used mathematical formalisms to answer these questions are ordinary differential equations (ODEs) and Boolean modeling.

Ordinary differential equations (ODEs) have been widely utilized to model signaling pathways. It is the most natural formalism for translating detailed reaction networks into a mathematical model. Indeed, equations can be directly derived using mass action laws, Michaelis-Menten kinetics or Hill functions for each reaction according to the observed behaviors. This framework has limitations, though. The first one concerns the difficulty to assign values to the kinetic parameters of the model. Ideally, these parameters would be extracted from experimental data. However, they are often chosen by the modeler so as to fit qualitatively the expected phenotypes. The second limitation concerns the cell population heterogeneity. In this case, ODEs are no longer appropriate since the approach is deterministic and thus focuses on the average behavior. To include non-determinism, an ODE model needs to be transformed into a stochastic chemical model. In this formalism, a master equation is written on the probabilities of the number of molecules for each species. In the translation process, the same parameters used in ODEs (more particularly in ODEs written with mass action law) can be used in the master equation, but in this case, the number of initial conditions explodes along with the computation time.

Boolean (or logical) formalism is another formalism used to model signaling pathways where genes/proteins are parameterized by 0s and 1s only. It is the most natural formalism to translate an influence network into a mathematical model. In such networks, each node corresponds to a species and each arrow to an interaction or an influence (positive or negative). In a Boolean model, a logical rule linking the inputs is assigned to each node. As a result, there are no real parameter values to adjust besides choosing the appropriate logical rules that best describe the system. In this paper, we will refer to a state in which each node of the influence network has a Boolean value as a network state, and the set of all possible transitions between the network states as a transition graph. There are two types of transition graphs, one deduced from the synchronous update strategy [1], for which all the nodes that can be updated are updated in one transition, and another one deduced from the asynchronous update strategy [2], for which only one node, of all the possible nodes, is updated in one transition. In the Boolean formalism, each transition can be interpreted as a “time” step, though this “time” does not characterize real biological time but rather an event. Stochasticity is an important aspect when studying cell populations. In Boolean framework, it can be applied: on nodes (by randomly flipping a node state [3,4]), on the logical rules (by allowing to change an AND gate into an OR gate [5]), and on the update rules (by defining the probability and the priority of changing one particular Boolean value before others in an asynchronous strategy [6] or by adding noise to the whole system in a synchronous strategy [7]). One of the main drawbacks of the Boolean approach is the explosion of solutions. In an asynchronous update strategy, the size of the transition graph can reach 2^#nodes^.

Both logical and continuous frameworks have advantages and disadvantages above-mentioned. We propose here to combine some of the advantages of both approaches in an algorithm that we call the “Boolean Kinetic Monte-Carlo” algorithm (BKMC). It consists of a natural generalization of the asynchronous Boolean dynamics [2], with a direct probabilistic interpretation. In BKMC framework, the dynamics is parameterized by a biological time and the order of update is noisy, which is less strict than priority classes introduced in GINsim [8]. A BKMC model is specified by logical rules as in regular Boolean models but with a more precise information: a numerical rate is added for each transition of each node.

BKMC is not intended to replace existing tools but rather to complement them. It is best suited to model signaling pathways in the following cases: 

• The model is based on an influence network, because BKMC is a generalization of the asynchronous Boolean dynamics. See “Examples” section. Note that this is a common requirement for most of Boolean software.

• The model describes processes for which information about the duration of a biological process is known, because in BKMC, time is parameterized by a real number. This is typically the case when studying developmental biology, where animal models provide time changes of gene/protein activities [9].

• The model describes heterogeneous cell population behavior, because BKMC has a probabilistic interpretation. For example, modeling heterogeneous cell population can help understand tissue formation based on cell differentiation [10].

• The model can contain many nodes (up to 64 in the present implementation), because BKMC is a simulation algorithm that converges fast. This can be useful for big models that have already been modeled with a discrete time Boolean method [11], in order to obtain a finer description of transient effects (see webpage for examples of published models: https://maboss.curie.fr).

Previous published works have also introduced a continuous time approach in the Boolean framework( [12-18]). In this article, we will first review some of these works and present BKMC algorithm. We will then describe the C++ software, MaBoSS, developed to implement BKMC algorithm and finally illustrate its use with three examples, a toy model, a published model of p53-MDM2 interaction and a published model of the mammalian cell cycle.

All abbreviations, definitions, algorithms and estimates used in this article can be found in Additional file 1. Throughout the article, all terms that are italicized are defined in the Additional file 1, “Definitions”.

## Results and discussion

### BKMC for continuous time Boolean model

#### Continuous time in Boolean modeling: past and present

In Boolean approaches for modeling networks, the state of each node of the network is defined by a Boolean value (node state) and the network state by the set of node states. Any dynamics in the transition graph is represented by sequences of network states. A node state is based on the sign of the input arrows and the logic that links them. The dynamics can be deterministic in the case of synchronized update [1], or non-deterministic in the case of asynchronized update [2] or probabilistic Boolean networks [7].

The difficulty to interpret the dynamics in terms of biological time has led to several works that have generalized Boolean approaches. These approaches can be divided in two classes that we call explicit and implicit time for discrete steps.

The explicit time for discrete steps consists of adding a real parameter to each node state. These parameters correspond to the time associated to each node state before it flips to another one ( [12,13]). Because data about these time lengths are difficult to extract from experimental studies, some works have included noise in the definition of these parameters [18]. The drawback of this method is that the computation of the Boolean model becomes sensitive to both the type of noise and the initial conditions. As a result, these time parameters become new parameters that need to be tuned carefully and thus add complexity to the modeling.

The implicit time for discrete steps consists of adding a probability to each transition of the transition graph in the case of non-deterministic transitions (asynchronous case). It is argued that these probabilities could be interpreted as specifying the duration of a biological process. As an illustration, let us assume a small network of two nodes, A and B. At time t, A and B are inactive: [AB] = [00]. In the transition graph, there exist two possible transitions at t+1: [00] → [01] and [00] → [10]. If the first transition has a significant higher probability than the second one, then we can conclude that B will have a higher tendency to activate before A. Therefore, it is equivalent to say that the activation of B is faster than the activation of A. Thus, in this case, the notion of time is implicitly modeled by setting probability transitions. In particular, priority rules, in the asynchronous strategy, consist of putting some of these probabilities to zero [6]. In our example, if B is faster than A then the probability of the transition [00] → [10] is zero. As a result, the prioritized nodes always activate before the others. From a different perspective but keeping the same idea, Vahedi and colleagues [14] have set up a method to deduce explicitly these probabilities from the duration of each discrete step. With the implementation of implicit time in a Boolean model, the dynamics remains difficult to interpret in terms of biological time.

As an alternative to these approaches, we propose BKMC algorithm.

#### Properties of BKMC algorithm

BKMC algorithm was built such as to meet the following principles: 

• The state of each node is given by a Boolean number (0 or 1), referred to as node state;

• The state of the network is given by the set of node states, referred to as network state;

• The update of a node state is based on the signs linking the incoming arrows of this node and the logic;

• Time is represented by a real number;

• Evolution is stochastic.

We choose to describe the time evolution of network states by a Markov process with continuous time, applied to the asynchronous transition graph. Therefore, the dynamics is defined by transition rates inserted in a master equation (see Additional file 1, “Basic information on Markov process”, section 1.1).

#### Markov process for Boolean model

Consider a network of *n* nodes (or agents, that can represent any species, *i.e.* mRNA, proteins, complexes, *etc.*). In a Boolean framework, the network state of the system is described by a vector **S** of Boolean values, *i.e.S*_*i*_∈{0,1},*i *= 1,…,*n* where *S*_*i*_ is the state of the node *i*. The set of all possible network states, also referred to as the network state space, will be called Σ.

A stochastic description of the state evolution is represented by a *stochastic processs*:*t *↦* s*(*t*) defined on t∈I⊂R applied on the network state space, where *I* is an interval: for each time t∈I⊂R, *s*(*t*) represents a random variable applied on the network state space. Thus, the probability of these random variables is written as:

(1) $$\begin{array}{rll} P\left[s(t)=S\right]\;\in & [0,1]\;\;\text{for any state}\;\;S\in \Sigma \; & \;\\ \text{with}\underset{S\in \Sigma }{\sum }P\left[s(t)=S\right]\;= & 1 \end{array}$$

Notice that for all *t*, *s*(*t*) are not independent, therefore $P\left[s(t)=S,s(t^{\prime })=S^{\prime }\right]\neq P\left[s(t)=S\right]P\left[s(t^{\prime })=S^{\prime }\right]$. From now on, we define $P\left[s(t)=S\right]$ as *instantaneous probabilities*. Since the instantaneous probabilities do not define the full stochastic process, all possible joint probabilities should also be defined.

In order to simplify the stochastic process, Markov property is imposed. It can be expressed in the following way: “the conditional probabilities in the future, related to the present and the past, depend only on the present” (see Additional file 1, “Basic information on Markov process”, section 1.1 for the mathematical definition). The formal definition of a *Markov process* is a stochastic process with the Markov property.

Any Markov process can be defined by (see Van Kampen [19], chapter IV): 

1. An initial condition:

(2) $$P\left[s(0)=S\right]\;;\forall S\in \Sigma$$

2. Conditional probabilities (of a single condition):

(3) $$P\left[s(t)=S|s(t^{\prime })=S^{\prime }\right]\;;\forall S,S^{\prime }\in \Sigma \;;\forall t^{\prime },t\in I;t^{\prime }<t$$

Concerning time, two cases can be considered: 

• If time is discrete: *t *∈* I *= {*t*_0_, *t*_1_,⋯}, it can be shown that all possible conditional probabilities are function of transition probabilities [20]: $P\left[s(t_{i})=S|s(t_{i-1})=S^{\prime }\right]$. In that case, a Markov process is often named a Markov chain.

• If time is continuous: *t *∈* I *= [*a*, *b*], it can be shown that all possible conditional probabilities are function of *transition rates*[19]: $\rho_{\left(S^{\prime }\to S\right)}(t)\in [0,\infty ]$.

Notice that a discrete time Markov process can be derived from continuous time Markov process, and is called a *Jump Process* with the following transition probabilities:

$$P_{S\to S^{\prime }}\equiv \frac{\rho_{S\to S^{\prime }}}{\underset{S^{\prime \prime }\in \Sigma }{\sum }\rho_{S\to S^{\prime \prime }}}$$

If the transition probabilities or transition rates are time independent, the Markov process is called a *time independent Markov process*. In BKMC, only this case will be considered. For a time independent Markov process, the *transition graph* can be defined as follows: a transition graph is a graph in Σ, with an edge between **S** and **S**^*′*^ if and only if $\rho_{S\to S^{\prime }}>0$ (or $P\left[s(t_{i})=S|s(t_{i-1})=S^{\prime }\right]>0$ if time is discrete).

#### Asynchronous Boolean dynamics as a discrete time Markov process

Asynchronous Boolean dynamics [2] is widely used in Boolean modeling. It can be easily interpreted as a discrete time Markov process [21,22] as shown below.

In the case of asynchronous Boolean dynamics, the system is given by *n* nodes (or agents), with a set of directed arrows linking these nodes and defining a network. For each node *i*, a Boolean logic *B*_*i*_(**S**) is specified and depends only on the nodes *j* for which there exists an arrow from node *j* to *i* (*e.g.B*_1_ =* S*_3_ AND NOT*S*_4_, where *S*_3_ and *S*_4_ are the Boolean values of nodes 3 and 4 respectively, and *B*_1_is the Boolean logic of node 1). The notion of *asynchronous transition* (AT) can be defined as a pair of network states $(S,S^{\prime })\in \Sigma$, written $\left(S\to S^{\prime }\right)$ such that

(4) $$\begin{array}{l} S_{j}^{\prime }=B_{j}(S)\;\text{for a given}\;j\;\\ S_{i}^{\prime }=S_{i}\;\text{for}\;i\neq j \end{array}$$

To define a Markov process, the transition probabilities $P\left[s(t_{i})=S|s(t_{i-1})=S^{\prime }\right]$ can be defined: given two network states **S** and **S**^*′*^, let *γ*(**S**) be the number of asynchronous transitions from **S** to all possible states **S**^*′*^. Then

(5) $$\begin{array}{l} \;P\left[s(t_{i})=S^{\prime }|s(t_{i-1})=S\right]=1/\gamma (S)\text{if}(S\to S^{\prime })\text{is an AT}\;\\ \;P\left[s(t_{i})=S^{\prime }|s(t_{i-1})=S\right]=0\text{if}(S\to S^{\prime })\text{is not an AT}\; \end{array}$$

In this formalism, the asynchronous Boolean dynamics completely defines a discrete time Markov process when the initial condition is specified. Notice that here the transition probabilities are time independent, *i.e.*$P\left[s(t_{i})=S|s(t_{i-1})=S^{\prime }\right]=P\left[s(t_{i+1})=S|s(t_{i})=S^{\prime }\right]$. Therefore, the approaches, mentioned in section “Continuous time in Boolean modeling: past and present”, that introduce time implicitly by adding probabilities to each transition of the transition graph, can be seen as a generalization of the definition of *γ*(**S**).

#### Continuous time Markov process as a generalization of asynchronous Boolean dynamics

To transform the discrete time Markov process described above in a continuous time Markov process, transition probabilities should be replaced by transition rates $\rho_{\left(S\to S^{\prime }\right)}$. In that case, conditional probabilities are computed by solving a master equation (equation 2 in Additional file 1, “Basic information on Markov process”, section 1.1). We present below the corresponding numerical algorithm, the *Kinetic Monte-Carlo* algorithm [23].

Because we want a generalization of the asynchronous Boolean dynamics, transition rates $\rho_{\left(S\to S^{\prime }\right)}$ are non-zero only if** S** and **S**^*′*^ differ by only one node. In that case, each Boolean logic *B*_*i*_(**S**)is replaced by two functions $R_{i}^{\text{up/down}}(S)\in [0,\infty [$. The transition rates are defined as follows: if *i* is the node that differs from **S** and **S**^*′*^, then

(6) $$\begin{array}{l} \rho_{\left(S\to S^{\prime }\right)}=R_{i}^{\mathrm{up}}(S)\text{if}\;S_{i}=0\;\\ \rho_{\left(S\to S^{\prime }\right)}=R_{i}^{\text{down}}(S)\text{if}\;S_{i}=1\; \end{array}$$

where *R*_*i*_^up^ corresponds to the activation rate of node *i*, and *R*_*i*_^down^ corresponds to the inactivation rate of node *i*. Therefore, the continuous Markov process is completely defined by all these *R*^up/down^ and an initial condition.

#### Asymptotic behavior of continuous time Markov process

In the case of continuous time Markov process, instantaneous probabilities always converge to a stationary distribution (see Additional file 1, “Basic information on Markov process”, corollary 2, section 1.2). A *stationary distribution* of a given Markov process corresponds to the set of instantaneous probabilities of a stationary Markov process which has the same transition probabilities (or transition rates) as the given discrete (or continuous) time Markov process. A *stationary Markov process* has the following property: for every joint probability $P\left[s(t_{1})=S^{\left(1\right)},s(t_{2})=S^{\left(2\right)},\ldots \right]$ and ∀*τ*:

(7) $$\begin{array}{lll} & P\left[s(t_{1})=S^{\left(1\right)},s(t_{2})=S^{\left(1\right)},\ldots \right]\; & \;\\ & \;\;=P\left[s(t_{1}+\tau )=S^{\left(1\right)},s(t_{2}+\tau )=S^{\left(1\right)},\ldots \right]\; \end{array}$$

Notice that instantaneous probabilities $P\left[s(t)=S\right]$ of a stationary stochastic process are time independent.

The asymptotic behavior of a continuous time Markov process can be detailed by using the concept of *indecomposable stationary distributions*: indecomposable stationary distributions are stationary distributions that cannot be expressed as a linear combination of different stationary distributions. A linear combination of stationary distributions is also a stationary distribution, since instantaneous probabilities are solutions of a master equation which is linear (see Additional file 1, “Basic information on Markov process”, equation 2, section 1.1). Therefore, a complete description of the asymptotic behavior is given by the linear combination of indecomposable stationary distributions to which the Markov process converges.

#### Oscillations and cycles

In order to describe a periodic behavior, the notion of cycle and oscillation for a continuous time Markov process is defined precisely.

A *cycle* is a loop in the transition graph. This is a topological characterization in the transition graph that does not depend on the exact value of the transition rates. It can be shown that a cycle with no outgoing edges corresponds to an indecomposable stationary distribution (see Additional file 1, “Basic information on Markov process”, corollary 1, section 1.2).

The question is then to link the notion of cycle to that of periodic behavior of instantaneous probabilities. The set of instantaneous probabilities cannot be perfectly periodic. They can display a damped oscillating behavior, or none at all (see Additional file 1, “Basic information on Markov process”, section 1.3). A *damped oscillatory* Markov process can be formally defined as a continuous time process that has at least one instantaneous probability with an infinite number of extrema.

According to theorems described in Additional file 1 (“Basic information on Markov process”, theorems 6-8 and Corollary 3, section 1.3), a necessary condition for having damped oscillations is that the transition matrix has at least one non-real eigenvalue (see Additional file 1, “Basic information on Markov process”, equation 4, section 1.1). In that case, there always exists an initial condition that produces damped oscillations. For the transition matrix to have a non-real eigenvalue, a Markov process needs to have a cycle. However, the reverse is not true: a Markov process with a cycle does not necessarily imply the existence of a non-real eigenvalue in the transition matrix. In the toy model of a single cycle, presented in the “Examples” section, non-real eigenvalues may or may not exist, according to different values of transition rates.

#### BKMC: Kinetic Monte-Carlo (Gillespie algorithm) applied to continuous time asynchronous Boolean Dynamics

It has been previously stated that a continuous time Markov process is completely defined by its initial condition and its transition rates. For computing any conditional probability (and any joint probability), a set of linear differential equations has to be solved (the master equation). Theoretically, the master equation can be solved exactly by computing the exponential of the transition matrix (see Additional file 1, “Basic information on Markov process”, equation 5, section 1.1). However, because the size of this transition matrix is 2^*n*^×2^*n*^, the computation soon becomes impossible if *n* is large. To remedy this problem, it is possible to use a simulation algorithm that samples the probability space by computing time trajectories in the transition graph.

The Kinetic Monte-Carlo [23] (or Gillespie algorithm [24]) is a simple algorithm for exploring the probability space of a Markov process defined by a set of transition rates. In fact, it can be understood as a formal definition of a continuous time Markov process. This algorithm produces a set of *realizations* or *stochastic trajectories* of the Markov process, given a set of uniform random numbers in [0,1]. By definition, a trajectory $\hat{S}(t)$ is a function from a time window [0,*t*_max_ to Σ. The set of stochastic trajectories represents the given Markov process in the sense that these trajectories can be used to compute probabilities. A finite set of these trajectories is produced, then, from this finite set, probabilities are estimated (as described in “Methods” section). The algorithm is based on an iterative step: from a state **S** at time *t*_0_(given two uniform random numbers), it produces a transition time *δt* and a new state $S^{\prime }$, with the following interpretation: the trajectory $\hat{S}(t)$ is such that $\hat{S}(t)=S$ for *t*∈*t*_0_*t*_0_ + *δt* and $\hat{S}(t_{0}+\mathrm{\delta t})=S^{\prime }$. Iteration of this step is done until a specified maximum time is reached. The initial state of each trajectory is based on the (probabilistic) initial condition that also needs to be specified.

The exact iterative procedure is the following. Given **S** and two uniform random numbers *u*,*u*^*′*^∈[0,1]: 

1. Compute the total rate of possible transitions for leaving state **S**: $\rho_{\text{tot}}\equiv \underset{S^{\prime }}{\sum }\rho_{\left(S\to S^{\prime }\right)}$.

2. Compute the time of the transition: $\mathrm{\delta t}\equiv -\log(u)/\rho_{\text{tot}}$

3. Order the possible new states ${S^{\prime }}^{\left(j\right)},j=1\ldots \;$ and their respective transition rates $\rho^{\left(j\right)}=\rho_{\left(S\to {S^{\prime }}^{\left(j\right)}\right)}$.

4. Compute the new state ${S^{\prime }}^{\left(k\right)}$ such that $\overset{k-1}{\underset{j=0}{\sum }}\rho_{j}<(u^{\prime }\rho_{\text{tot}})\leq \overset{k}{\underset{j=0}{\sum }}\rho_{j}$ (by convention, *ρ*^(0)^=0).

This algorithm will be referred to as *Boolean Kinetic Monte-Carlo* or BKMC.

### Practical use of BKMC, through MaBoSS tool

Biological data are translated into an influence network with logical rules associated to each node of the network. The value of one node depends on the value of the input nodes. For BKMC, another layer of information is provided when compared to the standard definition of Boolean models: transition rates are provided for all nodes, specifying the rates at which the node turns on and off. This refinement conserves the simplicity of Boolean description but allows to reproduce more accurately the observed biological dynamics. The parameters do not need to be exact as it is the case for nonlinear ordinary differential equation models, but they can be used to illustrate the relative speed of reactions. We developed a software tool, MaBoSS, that applies BKMC algorithm. MaBoSS stands for Markov Boolean Stochastic Simulator.

#### How to build a mathematical model using MaBoSS

Once MaBoSS is installed (see webpage for instructions, https://maboss.curie.fr), the protocol to follow to simulate a model can be described in four steps: 

1. Create the model using MaBoSS language in a file (myfile.bnd, for instance): (a) write the logic for each node, and (b) assign values to each transition rate.

2. Create the configuration file (myfile.cfg, for instance) to define the simulation parameters.

3. Run MaBoSS (the order of the arguments does not matter):

MaBoSS -c myfile.cfg -o myfile_out myfile.bnd

(we assume that MaBoSS is accessible through you PATH).MaBoSS creates three output files: 

• myfile_out_proptraj.csv

This file contains the network state probabilities on a time window, the entropy, the transition entropy and the Hamming distance distribution (see “Methods”)

• myfile_out_statdist.csv

This file contains the stationary distribution characterization (see “Methods”)

• myfile_out_run.txt

This file contains a summary of MaBoSS simulation run.

4. Import output csv files in Excel or R and generate your graphs.

#### Transition rates in MaBoSS

MaBoSS defines transition rates $\rho_{\left(S\to S^{\prime }\right)}$ by the functions *R*_*i*_^up/down^ (**S**) (see equations 6). The functions can be written using all Boolean operators (AND, OR, NOT, XOR), arithmetic operators (+,-,*,/), comparison operators and the conditional operator (?:). Examples of the use of the language are given below to illustrate three different cases: (1) different speeds for different inputs, (2) buffering effect and (3) the translation of discrete variables (with three values: 0, 1 and 2) into a Boolean model. 

1. Modeling different speeds for different inputs.Suppose that C is activated by A or B, but that B can activate C faster than A, and that C is inactivated when A and B are absent. In this case, we write: 

node C {

 rate_up = B ? $kb : (A ? $ka : 0.0);

 rate_down = !(A & B ) ? 1.0 : 0.0;

 }

 When C is off (equal to 0), it is activated by B at a speed $kb. If B is absent, then C is activated by A at a speed $ka. If both are absent, C is not activated. Note that if both A and B are present, because of the way the logic is written in this particular case, C is activated at the highest speed, the speed $kb. When C is on (equal to 1), it is inactivated at a rate equal to 1 in the absence of both A and B.

 To implement the synergistic effect of A and B, *i.e.* when both A and B are on, C is activated at a rate $kab, then we can write:

node C {

rate_up = (A & !B ? $ka : 0.0)+(B & !A ? $kb : 0.0) + (A & B ? $kab : 0.0);

 rate_down = !(A & B ) ? 1.0 : 0.0;

 }

2. Modeling buffering effect.Suppose that B is activated by A, but that B can remain active a long time after A has shut down. For that, it is enough to define different speeds of activation and inactivation:

node B {

rate_up = A ? 2.0 : 0.0;

rate_down = A ? 0.0 : 0.001;

}

 B is activated by A at a rate equal to 2. When A is turned off, B is inactivated more slowly at a rate equal to 0.001.

3. Modeling different levels for a given node.Suppose that B is activated by A, but if the activity of A is maintained, B can reach a second level. For this, we define a second node B_h (for “B high”) with the following rules:

node B {

rate_up = A ? 1.0 : 0.0; 

rate_down = (A | B_h) ? 0.0 : 1.0;

}

node B_h {

rate_up = (A & B) ? 1.0 : 0.0;

rate_down = (A) ? 0.0 : 1.0;

}

 In this example, B is separated in two variables: B which corresponds to the first level of B and B_h which corresponds to the higher level of B. B is activated by A at a rate equal to 1. If A disappears before B has reached its second level B_h then B is turned off at a rate equal to 1. If A is maintained and B is active, then B_h is activated at a rate equal to 1. When A is turned off, B_h is inactivated at a rate equal to 1.

#### Simulation parameters in MaBoSS

To simulate a model in MaBoSS, a set of parameters needs to be adjusted (see “Parameter list” in the reference card available in the webpage). MaBoSS assigns default values, however, they need to be tuned for each model to achieve optimal performances: the best balance between the convergence of estimates and the computation time needs to be found. Therefore, several simulations should be run with different sets of parameters for best tuning. 

• Internal nodes: *node.is_internal*As explained in “Methods” (in “Initial conditions and outputs”), internal nodes correspond to species that are not measured explicitly. Practically, the higher the number of internal nodes, the better the convergence of the BKMC algorithm.

• Time window for probabilities: *timetick*This parameter is used to compute estimates of network state probabilities (see “Network state probabilities on a time window” in “Methods”). A time window can be set as the minimum time needed for nodes to change their states. This parameter also controls the convergence of probability estimates. The larger the time window, the better the convergence of probability estimates.

• Maximum time: *max_time*MaBoSS produces trajectories for a predefined amount of time, set by the parameter max_time. If the time of the biological process is known, then the maximum time parameter can be explicitly set. If the time of the biological process is not known, then there exists a more empirical way to set the maximum time. It is advised to choose a maximum time parameter that is slightly bigger than the inverse of the smallest transition rate.Note that the computing time in MaBoSS is proportional to this maximum time. Moreover, the choice of the maximum time impacts the stationary distribution estimates: a longer maximum time increases the quality of these estimates.

• Number of trajectories: *sample_count*This parameter directly controls the quality of BKMC estimation algorithm. Practically, the convergence of the estimates increases as the number of trajectories is increased.

• Number of trajectories (*statdist_traj_count*) and similarity threshold (*statsdist_cluster_threshold*) for stationary distribution estimatesThe *statdist_traj_count* parameter corresponds to a subset of trajectories used only for stationary distribution estimates. To avoid explosion of computing time, this parameter needs to be lower than the number of trajectories (rather than equal to).The *statsdist_cluster_threshold* parameter corresponds to the threshold for constructing the clusters of stationary distribution estimates. Ideally, it should be set to a high value (close to 1). However, if the threshold is too high then the clustering algorithm might not be efficient.

#### Comparison with biological data

Each node of the network should account for different levels of activity of the corresponding species (mRNA, protein, protein complex, etc.). It is possible to have more than two levels for one node, as shown in the example “Modeling different levels for a given node”.

It is possible to extract the transition rates from experimental data, using the following property: the rate of a given transition is the inverse of the mean time for this transition to happen. It should be noticed than BKMC is an algorithm based on a linear equation (Additional file 1, “Basic information on Markov process”, equation 2, section 1.1); therefore, small variations of transition rates will not affect the qualitative behavior of the model.

BKMC algorithm provides estimates of the network state probabilities over time. These probabilities can be interpreted in terms of a cell population. The asymptotic behavior of a model, represented by a linear combination of indecomposable stationary distributions, can be interpreted as a combination of cell sub-populations. Indeed, a sub-population can be defined by network states with non-zero probability in the indecomposable stationary distribution. Therefore, a cell in a sub-population can only evolved in this sub-population (Additional file 1, “Basic information on Markov process”, corollary 1, section 1.2 and from the definition of strongly connected component with no outgoing edges).

#### Comparison of MaBoSS with other existing tools for qualitative modeling

**Figure 1:**
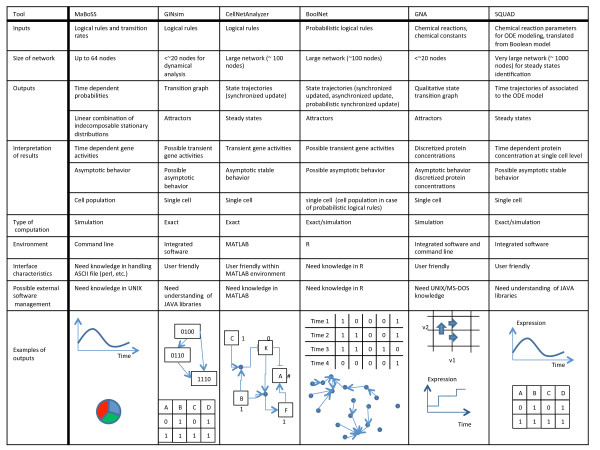
**Comparison of tools for discrete modeling, biological implication.** Comparison table of the following tools: MaBoSS, GINsim, CellNetAnalyzer, BoolNet, GNA, SQUAD. Technical aspects are provided, along with the inputs/outputs relations between a model and data. The last row illustrates graphically the typical outputs that can be obtained from each tool.

MaBoSS contributes to the effort of tool development for qualitative modeling of biological networks. We propose to compare MaBoSS to some existing tools. However, it is difficult to compare the performance of these tools since each of them achieves different purposes and provides different outputs. As an alternative, we recapitulate, in Figure 1, the characteristics and implications for each software. Some tools may be more appropriate than others according to the type of input, network size and expected output. Figure 1 is intended to help the users decide which software to use in a practical situation. We consider the following tools: GINsim [8], CellNetAnalyzer [25], BoolNet [26], GNA [27], and SQUAD [28]. This list is not exhaustive but informs on where MaBoSS stands.

As an illustration, the third example of the “Examples” section below, the mammalian cell cycle, was implemented in three of the tools presented in Figure 1: MaBoSS, GINsim, BoolNet (see Additional file 2 “Model of the mammalian cell cycle with GINsim, BoolNet and MaBoSS.” for details of the results).

### Examples

We have applied BKMC algorithm to three models of different sizes. The first one is a toy model illustrating the dynamics of a single cycle; the second one is a published Boolean model of p53-Mdm2 response to DNA damage and illustrates a multi-level case; and the third one is a published Boolean model of mammalian cell cycle regulation. Note that MaBoSS has been used for these three examples, but Markov process can be computed directly for the two first ones, without our BKMC algorithm because these models are small enough (by computing exponential of transition matrix, see Additional file 1, “Basic information on Markov process”, section 1.1), as proposed in [16]. BKMC is best suited for larger networks, when the network state space is too large to be computed with standard existing tools (>∼2^10^). The first two examples were chosen for their simplicity, and because they illustrate how global characterizations (entropy and transition entropy, see “Entropiesł::bel sect:entropies” in “Methods”) can be used. The third example shows the use of BKMC/MaBoSS for a more consequent and complex model for which the analysis is not obvious.

For the purpose of this article, we built the transition graphs for the first two examples (with GINsim [8]) in order to help the reasoning. However, it is important to note that BKMC algorithm does not construct the transition graph explicitly.

All input files and results are given in the webpage of MaBoSS ( https://maboss.curie.fr) with additional examples.

#### Toy model of a single cycle

**Figure 2:**
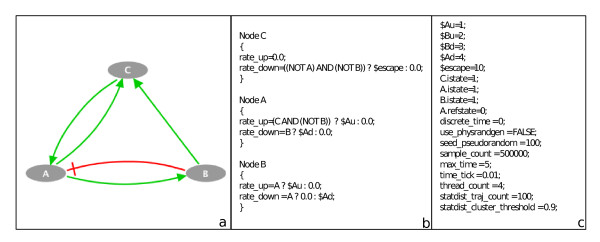
**Toy model.** Toy model of a single cycle. **(a)** Influence network. **(b)** Logical rules and transition rates of the model. **(c)** Simulation parameters.

We consider three species, A, B and C, where A is activated by C and inhibited by B, B is activated by A and C is activated by A or B (Figure 2a).

**Figure 3:**
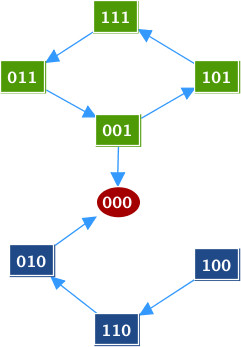
**Transition graph of the toy model.** Transition graph for the toy model (generated by GINsim). The node states should be read as [ABC] = [^∗∗∗^]. [ABC]=[100] corresponds to a state in which only A is active. The nodes in green belong to a cycle, the node in red is the fixed point and the other nodes are in blue.

The model is defined within the language of MaBoSS by a set of logical rules associated to each node (Figure 2b) and simulation parameters set for optimal performances (Figure 2c). The associated transition graph, generated by GINsim, is shown in Figure 3.

The only stationary distribution is the fixed point [ABC]=[000]. We study two cases: when the rate of the transition from state [001] to state [000] (corresponding to the inactivation of C) is fast and when this rate is slow. We will refer to this transition rate as the *escape rate*. For both cases, we plot the time trajectories of the probabilities of the fixed point [ABC]=[000] and of the probabilities of A active [ABC]=[1^∗∗^] where ^∗^can be either 1 or 0, along with the trajectories of the entropy and the transition entropy.

**Figure 4:**
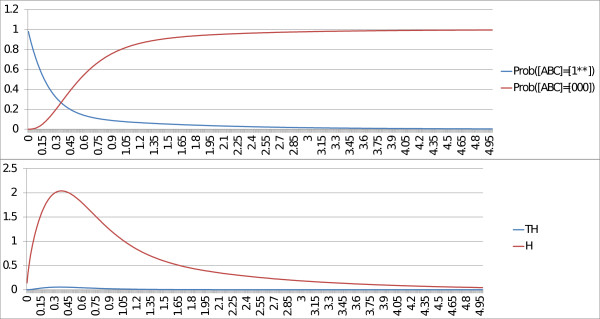
**MaBoSS outputs of the toy model with fast escape rate.** BKMC algorithm is applied to the toy model, with a fast escape rate. Trajectory of the network state probabilities [ABC]=[000] and [ABC]=[1^∗∗^] (where ^∗^can be either 0 or 1), the entropy (*H*) and the transition entropy (*TH*) are plotted. Because the probability of [ABC]=[000] converges to 1, [ABC]=[000] is a fixed point. The asymptotic behavior of both the entropy and the transition entropy is also the signature of a fixed point.

In the first case, when the escape rate is fast, we set the parameter for the transition to a high value (rate_up = 10). In Figure 4, we notice that the probability that [ABC] is equal to [000] converges to 1. We can conclude that [ABC]=[000] is a fixed point. In addition, the entropy and the transition entropy converge to 0. With BKMC, these properties confirm that [ABC]=[000] is a fixed point. The peak in the trajectory of the entropy (between times 0 and 0.6) corresponds to a set of states that are transiently activated before reaching the fixed point.

**Figure 5:**
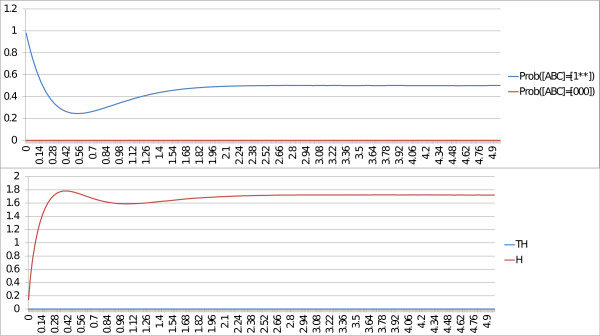
**MaBoSS outputs of the toy model with slow escape rate.** BKMC algorithm is applied to the toy model, with a slow escape rate. Trajectory of the network state probabilities [ABC]=[000] and [ABC]=[1**], the entropy (*H*) and the transition entropy (*TH*) are plotted. The asymptotic behavior of both the entropy and the transition entropy seems to be the signature of a cycle.

**Figure 6:**
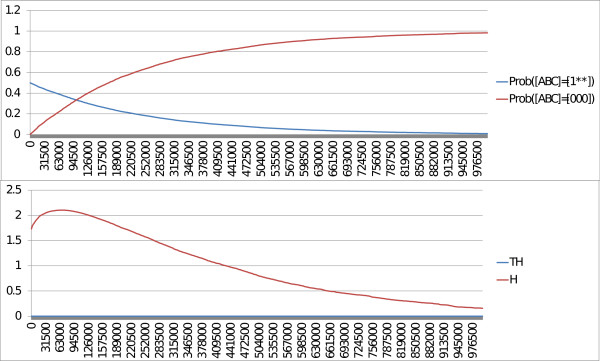
**MaBoSS outputs of toy model with slow escape rate, large time scale.** BKMC algorithm is applied to the toy model, with a slow escape rate, plotted on a larger time scale. Trajectory of probabilities ([ABC]=[000] and [ABC]=[1**]), the entropy (*H*) and the transition entropy (*TH*) are plotted. On a large time scale, the asymptotic behavior of both the entropy and the transition entropy is similar to the case of the fast escape rate (Figure 3).

In the second case, when the escape rate is slow, we set the parameter for the transition to a low value (rate_down = 10^−5^). As illustrated in Figure 5, the transition entropy is and remains close to zero but the entropy does not converge to zero, which is the signature of a cyclic stationary distribution (see “Entropiesł::bel sect:entropies” in “Methods”). This corresponds to the cycle [111] → [011] → [001] → [101] in the transition graph (Figure 3). However, as seen in the transition graph, one state in the cycle has an outgoing edge that leads to the fixed point (through the transition [001] → [000] in Figure 3). If the trajectories are plotted on a larger time scale (Figure 6), the entropy eventually converges to 0 and the trajectory of the fixed point converges to 1, which corresponds to the case of fast escape rate. Since the value of the transition entropy of Figure 5 is not exactly zero, but 10^−4^, it can be anticipated that the cyclic behavior is not stable. We can conclude on stable cyclic behaviors only when the transition entropy is exactly 0.

By considering the spectrum of the transition matrix (see Additional file 1, “Basic information on Markov process”, section 1.1 and proof of theorem 4), it can be proven that the model with a slow escape rate is a damped oscillatory process whereas the model with a large escape rate is not. As mentioned previously, a cycle in the transition graph may or may not lead to an oscillatory behavior. Moreover, if the transition entropy seems to converge to a small value on a small time scale, and the entropy does not, this behavior illustrates the case of a transient cycle in the transition graph.

#### p53-Mdm2 signaling

**Figure 7:**
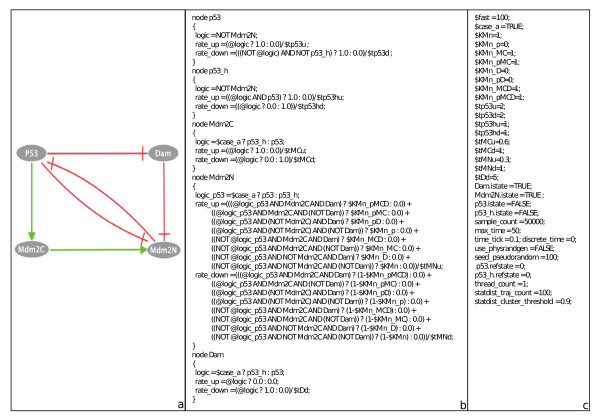
**Model of p53 response to DNA damage.** Model of p53 response to DNA damage. **(a)** Influence network. **(b)** Logical rules and transition rates of the model. **(c)** Simulation parameters.

We consider a model of p53 response to DNA damage [18]. p53 interacts with Mdm2, which appears in two forms, cytoplasmic and nuclear. On one hand, p53 upregulates the level of cytoplasmic Mdm2 (Mdm2c), which is then transported into the nucleus, and inhibits the export of nuclear Mdm2 (Mdm2n). On the other hand, nuclear Mdm2 (Mdm2n) facilitates the degradation of p53 through ubiquitination. In the model, stress regulates the level of DNA damage (Dam), which in turn participates in the degradation process of Mdm2 in the nucleus. p53 inhibits DNA damage signal by promoting DNA repair. Here, stress is not shown explicitly (Figure 7a).

**Figure 8:**
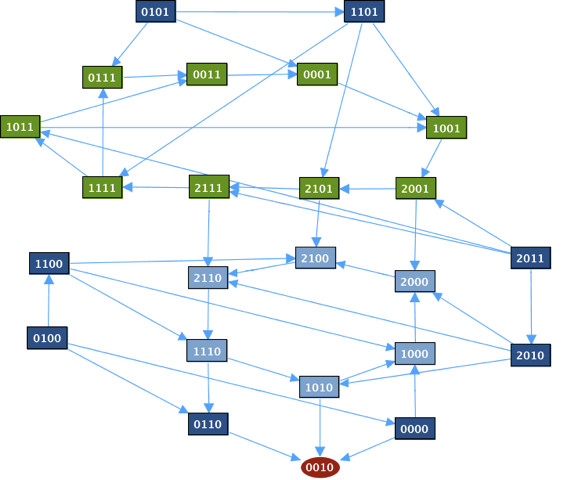
**Transition graph of the model of p53 response to DNA damage.** Transition graph of the p53 model (generated by GINsim). The node states should be read as [p53 Mdm2C Mdm2N Dam] = [^∗∗∗∗^] (where ^∗^can be either 0 or 1). For instance, [p53 Mdm2C Mdm2N Dam]=[1000] corresponds to a state in which only p53 (at its level 1) is active. The nodes in green and the nodes in light blue belong to two cycles, the node in red is the fixed point and the other nodes are in dark blue.

The model is written in MaBoSS, with two levels of p53 (Figure 7b), as it is done in Abou-Jaoudé *et al.*[18] with the appropriate simulation parameters (Figure 7c). The associated transition graph, also generated by GINsim, is given in Figure 8. It shows the existence of two cycles and of a fixed point [p53 Mdm2C Mdm2N Dam] = [0010] where nuclear Mdm2 is on and the rest is off.

**Figure 9:**
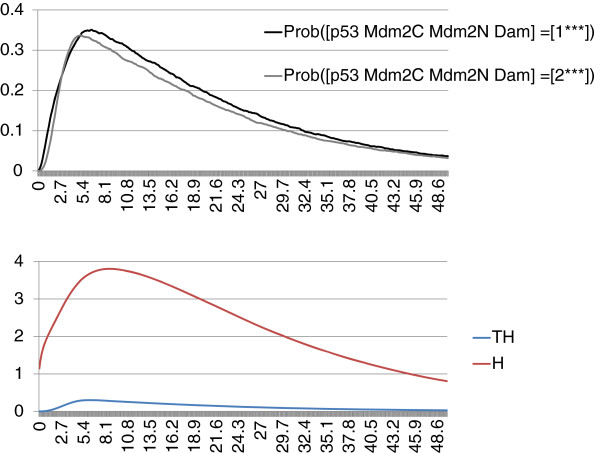
**MaBoSS outputs of the model of p53 response to DNA damage.** Trajectories of the network state probabilities of [p53 Mdm2C Mdm2N Dam] = [1^∗∗∗^] and of [p53 Mdm2C Mdm2N Dam] = [2^∗∗∗^], the entropy (*H*) and the transition entropy (*TH*) are plotted.

In order to represent the activity of p53, the trajectories of the probabilities of all network states with p53 equal to 1 and with p53 equal to 2 are plotted (Figure 9, upper panel), with the initial condition: [p53 Mdm2C Mdm2N Dam] = [0^∗^11] and for the situation when p53 is set to its highest value (2 equivalent to p53_h) and thus can promote Mdm2 cytoplasmic activity.

The qualitative results obtained with MaBoSS are similar to those of Abou-Jaoudé and colleagues. However, at the level of cell population, some discrepancies appear: in Figure 9, no damped oscillations can be seen as opposed to Figure 8 of their article. The reason is that, in their computations, the noise imposed on time is defined by a square distribution on a limited time frame, whereas in BKMC, Markovian hypotheses imply that the noise distribution is more spread out from 0 to infinity. The consequence is that synchronization is lost very fast. Damped oscillations could be observed with BKMC with a particular set of parameters: fast activation of p53 and slow degradation of p53 (results not shown).

With MaBoSS, we clearly interpret the system as a population and not as a single cell. In addition, we can simulate different contexts, presented in the initial article as different models, within one single model that uses different simulation parameters to account for these contexts.

Note that the existence of transient cycles, as shown in the toy model, can be deduced from the trajectory of the entropy that is significantly higher than the trajectory of the transition entropy (which is non-zero, therefore the transient cycles are not stable) (Figure 9, lower panel).

#### Mammalian cell cycle

For the last example, we propose a model of the mammalian cell cycle initially published as on ODE model by Novák and Tyson [29] and translated into a Boolean model by Fauré and colleagues [6]. The latter model encompasses 10 nodes, which describe the mechanisms controlling the activity of the different CDK/cyclin complexes, the main actors of cell cycle regulation and the dynamics of entry into the cell cycle in presence of growth factors.

We implement the logical rules of the published model in MaBoSS and define two parameter values for the transition rates: a slow one (set to 1) and a fast one (set to 10). The choice between slow and fast rates for each transition is based on the choice made in the published Boolean model: different priority classes were used in mixed discrete a/synchronous simulation and corresponded to the differences in speed of cellular processes such as transcription, degradation and protein modification. We could, of course, refine the analysis by setting different rates for each transition. The network, the logical rules and the simulation parameters can be found on the webpage.

As mentioned before, MaBoSS can provide two types of outputs: the probabilities of different network states over time (along with the entropy and transition entropy) and the indecomposable stationary distributions.

**Figure 10:**
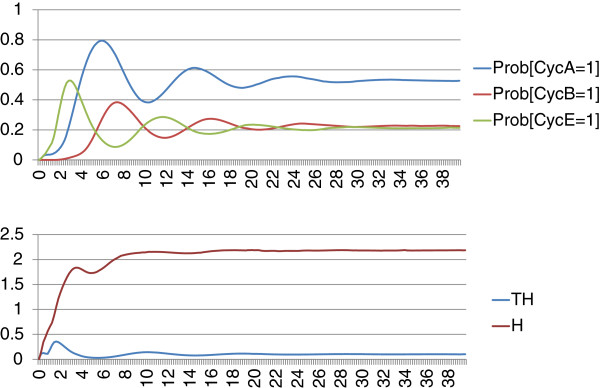
**MaBoSS outputs of the model of the mammalian cell cycle: trajectories of probabilities.** BKMC algorithm is applied to the mammalian cell cycle model, with an initial condition corresponding to a G1 state in the presence of growth factors (CyclinD is on). Trajectories of the cyclins probabilities, the entropy (*H*), transition entropy (*TH*) are plotted. The asymptotic behavior corresponds to the first indecomposable stationary distribution identified in Figure 10.

We consider two biological cases, in the presence of growth factors where the cell enters its division cycle and in the absence of growth factors where the cell is stuck in a G1-like state (state preceding replication of DNA). In the model, the activity of CyclinD (CycD), a G1-cyclin, illustrates the presence of growth factors. In our simulations, we set an initial condition corresponding to a G1 state with two CDK/cyclin inhibitors, p27 and cdh1, on, and with CyclinD on in order to account for the external growth signal. We plot the trajectories of the probabilities of all the cyclins A, B and E (Figure 10, upper panel). The cyclins’ activities exhibit an oscillatory behavior. Each oscillation can be interpreted as a cell division cycle. However, these oscillations are damped. This can be explained by the fact that these probabilities should be interpreted at the cell population level and after few cycles, the cells become desynchronized. Moreover the trajectories of the entropy and the transition entropy exhibit the signature of cyclic attractors (Figure 10, lower panel).

**Figure 11:**
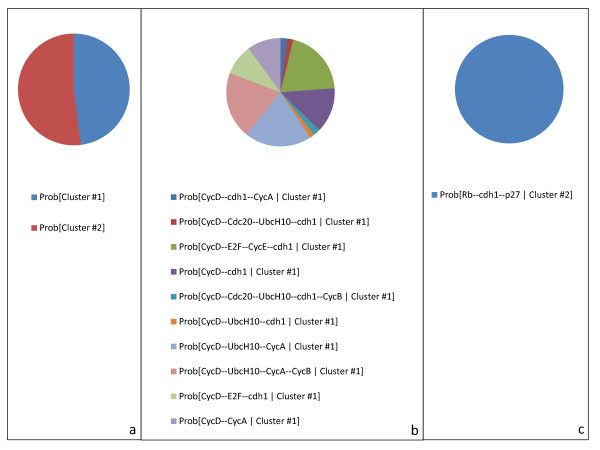
**MaBoSS outputs of the model of the mammalian cell cycle: stationary distributions.** BKMC algorithm is applied to the mammalian cell cycle model, with random initial conditions. Results of the clustering algorithm that associates a cluster to each indecomposable stationary distribution. **(a)** Probability of reaching each identified cluster; these probabilities are estimated by the proportion of trajectories that belong to each cluster. **(b)** First estimated cluster that can be interpreted as a desynchronized population of cells that are dividing. **(c)** Second estimated cluster, corresponding to a fixed point, that can be interpreted as a G1 cell cycle arrest with no growth factors.

The indecomposable stationary distributions are identified by the clustering algorithm of MaBoSS and illustrated in Figure 11. The two clusters in Figure 11a show the two types of solutions for random initial conditions: one multi-cyclic solution when CyclinD is on, and which corresponds to the distribution of network states of the asymptotic solution of Figure 11b, and one fixed point corresponding to a G1 arrest when CyclinD is off (Figure 11c).

These two indecomposable stationary distributions correspond to the two attractors identified by discrete time modeling in Fauré *et al.* In the discrete time algorithm, the asymptotic behavior is described in terms of attractors (sub-parts of the transition graph); in our algorithm, the asymptotic behavior is described in terms of network state probability distributions.

## Conclusions

We have presented a new algorithm, Boolean Kinetic Monte-Carlo or BKMC, applicable to dynamical simulation of signaling networks based on continuous time in the Boolean framework. BKMC algorithm is a natural generalization of the asynchronous Boolean dynamics [2], with time trajectories that can be interpreted in terms of biological time. The variables of the Boolean model represent biological species and the parameters represent rates of activation or inactivation of these species that, ideally, could be deduced from experimental data.

We applied this algorithm to three different models: a toy model that illustrates a simple cyclic behavior, a published model of p53 response to DNA damage, and a published model of mammalian cell cycle dynamics.

This algorithm is provided within a freely available software, MaBoSS, that can run BKMC algorithm on networks up to 64 nodes in the present version. The construction of a model uses a specific language that introduces logical rules and transition rates of node activation/inactivation in a flexible manner. The software provides global and semi-global outputs of the model dynamics that can be interpreted as signatures of the dynamical behaviors. These interpretations become particularly useful when the network state space is too large to be handled. The convergence of BKMC algorithm can be controlled by tuning some simulation parameters: maximum time of the simulation, number of trajectories, length of a time window on which the average of probabilities is performed, and the threshold for the definition of stationary distribution clusters.

The next step is to apply BKMC algorithm with MaBoSS on other existing large signaling networks, *e.g.* EGFR pathway [30], the apoptosis pathway [31], etc. The translation of existing Boolean models in MaBoSS is straightforward but requires the addition of transition rates. In these future works, we expect to illustrate the advantage of BKMC on other simulation algorithms. Moreover, in future developments of MaBoSS, we plan to introduce methods for sensitivity analyses, refine approximation methods used in BKMC, and generalize Markov property.

We also expect to implement MaBoSS in broadly used software environments for Boolean modeling, like GINsim [8] or CellNetAnalyzer [25].

## Methods

BKMC generates stochastic trajectories. In this section, we describe how we use and interpret these trajectories.

### 

#### Network state probabilities on a time window

To relate continuous time probabilities to real processes, an observable time window *Δt* is defined. A discrete time ( τ∈N) stochastic process *s*(*τ*) (that is not necessary Markovian) can be extracted from the continuous time Markov process:

(8) $$P\left[s(\tau )=S\right]\equiv \frac{1}{\Delta t}\int_{\tau \Delta t}^{(\tau +1)\Delta t}\text{dt}\;P\left[s(t)=S\right]$$

BKMC is used for estimating $P\left[s(\tau )=S\right]$ as follows: 

1. **Estimate for one trajectory.** For each trajectory *j*, compute the time for which the system is in state **S**, in the window [*τΔt*,(*τ* + 1)*Δt*]. Divide this time by *Δt*. Obtain an estimate of $P\left[s(\tau )=S\right]$ for trajectory *j*, *i.e.*${\hat{P}}_{j}\left[s(\tau )=S\right]$.

2. **Estimate for a set of trajectories.** Compute the average over *j* of all ${\hat{P}}_{j}\left[s(\tau )=S\right]$ to obtain $\hat{P}\left[s(\tau )=S\right]$. Compute the error of this average ( $\sqrt{\text{Var}(\hat{P}\left[s(\tau )=S\right])/\text{\# trajectories}}$).

#### Entropies

Once $P\left[s(\tau )=S\right]$ is computed, the entropy *H*(*τ*) can be estimated:

(9) $$H(\tau )=-\underset{S}{\sum }\log_{2}\left(P\left[s(\tau )=S\right]\right)P\left[s(\tau )=S\right]$$

The entropy measures the disorder of the system. Maximum entropy means that all states have the same probability; a zero entropy means that one of the states has a probability of one. The estimation of the entropy can be seen as a global characterization of a full probability distribution by a single real number. The choice of $\log_{2}$ allows the interpretation of *H*(*τ*) in an easier manner: 2^*H*(*τ*)^is an estimate of the number of states that have a non-negligible probability in the time window [*τΔt*,(*τ* + 1)*Δt*]. A more computer-like interpretation of *H*(*τ*) is the number of bits that are necessary for describing states of non-negligible probability.

The *Transition EntropyTH* is a finer measure that characterizes the system at the level of a single trajectory. It can be computed in the following way: for each state **S**, there exists a set of possible transitions $S\to S^{\prime }$. For each of these transitions, a probability is associated:

(10) $$P_{S\to S^{\prime }}\equiv \frac{\rho_{S\to S^{\prime }}}{\underset{S^{\prime \prime }}{\sum }\rho_{S\to S^{\prime }}}.$$

By convention, $P_{S\to S^{\prime }}=0$ if there is no transition from **S **to any other state.

Therefore, the transition entropy *TH* can be associated to each state **S**:

(11) $$\text{TH}(S)=-\underset{S^{\prime }}{\sum }\log_{2}(P_{S\to S^{\prime }})P_{S\to S^{\prime }}$$

Similarly, *TH*(**S**) = 0 if there is no transition from **S** to any other state. The transition entropy on a time window *TH*(*τ*) is defined as:

$$\text{TH}(\tau )=\underset{S}{\sum }P\left[s(\tau )=S\right]\text{TH}(S)$$

This transition entropy is estimated in the following way: 

1. **Estimate for one trajectory.** For each trajectory *j*, compute the set Φ of visited states **S** in the time window [*τΔt*,(*τ* + 1)*Δt*] and their respective duration *μ*_**S**_. The estimated transition entropy is:

(12) $${\hat{\text{TH}(\tau )}}_{j}=\underset{S\in \Phi }{\sum }\text{TH}(S)\frac{\mu_{S}}{\Delta t}$$

2. **Estimate for a set of trajectories.** Compute the average over *j* of all ${\hat{\text{TH}(\tau )}}_{j}$ to obtain $\hat{\text{TH}(\tau )}$. Compute the error of this average ( $\sqrt{\text{Var}(\hat{\text{TH}(\tau )})/\text{\# trajectories}}$).

This transition entropy is a way to measure how deterministic the dynamics is. If the transition entropy is always zero, the system can only make a transition to a given state.

If probability distributions on a time window tend to constant values (or tend to a stationary distribution), the entropy and the transition entropy can help characterize this stationary distribution such that: 

• A fixed point has zero entropy and zero transition entropy,

• A cyclic stationary distribution has non-zero entropy and zero transition entropy.

Entropy and transition entropy can be considered as “global characterizations” of the model: for a given time window, they always consist of two real numbers, whatever the size of the network is.

#### Hamming distance distribution

The *Hamming Distance* between two states **S**and **S**^*′*^ is the number of nodes that have different node states between **S**and **S**^*′*^:

(13) $$\text{HD}(S,S^{\prime })\equiv \underset{i}{\sum }(1-\delta_{S_{i},S_{i}^{\prime }})$$

where *δ* is the Kronecker delta ( $\delta_{S_{i},S_{i}^{\prime }}=1$ if *S*_*i *_=* S*_*i*_^*′*^, $\delta_{S_{i},S_{i}^{\prime }}=0$ if *S*_*i *_≠* S*_*i*_^*′*^). Given a reference state **S**_ref_, the Hamming distance distribution (over time) is given by:

(14) $$P(\text{HD},t)=\underset{S}{\sum }P\left[s(t)=S\right]\delta_{\text{HD},\text{HD}(S,S_{\text{ref}})}$$

The estimation of the Hamming distance distribution on a time window **P**(*HD*,*τ*) is similar to that of stochastic probabilities on a time window.

The Hamming distance distribution is a useful characterization when the set of instantaneous probabilities is compared to a reference state (**S**_ref_). In that case, the Hamming distance distribution describes how far this set is to this reference state. The Hamming distance distribution can be considered as a “semi-global” characterization of time evolution: for a given time window, the size of this characterization is the number of nodes (to be compared with probabilities on a time window whose size is 2^#nodes^).

#### Input, internal, output and reference nodes

*Input Nodes* are defined as the nodes for which the initial condition is fixed. Therefore, each trajectory of BKMC starts with fixed values of input nodes and random values of other nodes.

*Internal nodes* are nodes that are not considered for computing probability distributions, entropies and transition entropies. *Output nodes* are nodes that are not internal. Technically, probabilities are summed up over network states that differ only by the state of internal nodes. These internal nodes are only used for generating time trajectories with BKMC algorithm. Usually, nodes are chosen to be internal when the corresponding species is not measured experimentally. Mathematically, it is equivalent to transform the original Markov process to a new stochastic process (that is not necessary Markovian) defined on a new network state space. This new state space is defined by the states of the output nodes. This raises the question of the transition entropy *TH*: formally, this notion has only a sense within Markovian processes, *i.e.* when there are no internal nodes. Here, we generalize the notion of transition entropy even in the case of internal nodes. Suppose that the system is in state **S**: 

• If the only possible transitions from state **S **to any other state consist of flipping an internal node, the transition entropy is zero.

• If there is, at least, one transition from state **S **to another state that flips an output node, then only the output nodes will be considered for computing probabilities in equation 10. In particular, $\underset{S^{\prime }}{\sum }\rho_{S\to S^{\prime }}$ is computed only on output node flipping events.

*Reference nodes* are nodes for which a reference node state is specified and for which the Hamming distance is computed. In this framework, a reference state is composed of reference nodes for which the node state is known and non-reference nodes for which the node state is unknown. Note that non-reference nodes may differ from internal nodes.

#### Stationary distribution characterization

It can be shown (see Additional file 1, “Basic information on Markov process”, corollary 2, section 1.2) that instantaneous probabilities of a continuous time Markov process converge to a stationary distribution. Fixed points and cycles are two special cases of stationary distributions. They can be identified by the asymptotic behavior of entropy and transition entropy (this works only if no nodes are internal): 

• If both the transition entropy and the entropy converge to zero, then the process converges to a fixed point.

• if the transition entropy converges to zero and the entropy does not, then the process converges to a cycle.

More generally, the complete description of the Markov process asymptotic behavior can be expressed as a linear combination of the indecomposable stationary distributions.

A set of finite trajectories, produced by BKMC, can be used to estimate the set of indecomposable stationary distributions. Consider a trajectory $\hat{S}(t),t\in [0,T],i=1,\cdots \;,n$. Let $I_{S}(t)\equiv \delta_{S,\hat{S}(t)}$. The estimation of the associated indecomposable stationary probability distribution (*s*_0_) is done by averaging over the whole trajectory:

(15) $$\hat{P}\left[s_{0}=S\right]=\frac{1}{T}\int_{0}^{T}\text{dt}I_{S}(t)$$

Therefore, a set of indecomposable stationary distribution estimates can be obtained by a set of trajectories. These indecomposable stationary distribution estimates should be clustered in groups, where each group consists of estimates for the same indecomposable stationary distribution. For that, we use the fact that two indecomposable stationary distributions are identical if they have the same support, *i.e.* the same set of network states with non-zero probabilities (shown in Additional file 1, “Basic information on Markov process”, theorem 2, section 1.2). Therefore, it is possible to quantify how similar two indecomposable stationary distribution estimates are. A *similarity coefficient*$D(s_{0}^{\left(i\right)},s_{0}^{\left(j\right)})\in [0,1]$, given two stationary distribution estimates *s*_0_^(*i*)^ and *s*_0_^(*i*)^ , is defined:

(16) $$\begin{array}{l} D(s_{0}^{\left(i\right)},s_{0}^{\left(j\right)})\equiv \left(\underset{S\in \text{support}(s_{0}^{\left(i\right)},s_{0}^{\left(j\right)})}{\sum }\hat{P}\left[s_{0}^{\left(i\right)}=S\right]\right)\;\\ \;\;\;\times \left(\underset{S^{\prime }\in \text{support}(s_{0}^{\left(i\right)},s_{0}^{\left(j\right)})}{\sum }\hat{P}\left[s_{0}^{\left(j\right)}=S^{\prime }\right]\right)\; \end{array}$$

where

(17) $$\begin{array}{ll} \text{support}(s_{0}^{\left(i\right)},s_{0}^{\left(j\right)})\equiv \left\{S\text{such that}\hat{P}\left[s_{0}^{\left(i\right)}=S\right]\right)\; & \;\\ \;\;\;\;\left(\times \hat{P}\left[s_{0}^{\left(j\right)}=S\right]>0\right\}\; \end{array}$$

Clusters can be constructed when a similarity threshold *α* is provided. A cluster of stationary distributions is defined as follows:

(18) $$C=\left\{s_{0}|\;\exists s_{0}^{\prime }\in C\text{s. t.}D(s_{0},s_{0}^{\prime })\geq \alpha \right\}$$

For each cluster C, a distribution estimate $s_{C}$, associated to an indecomposable stationary distribution, can be defined:

(19) $$P\left[s_{C}=S\right]=\frac{1}{\left|C\right|}\underset{s\in C}{\sum }P\left[s=S\right]$$

Errors on this estimate can be computed by:

(20) $$\text{Err}\left(P\left[s_{C}=S\right]\right)=\sqrt{\text{Var}(P\left[s=S\right],s\in C)/|C|}$$

Notice that this clustering procedure has no sense if the process is not Markovian; therefore, no nodes are considered as internal.

## Abbreviations

BKMC: Boolean Kinetic Monte-Carlo; AT: Asynchronous transition; ODEs: Ordinary differential equations; MaBoSS: Markov Boolean Stochastic Simulator.

## Competing interests

The authors declare that they have no competing interests.

## Authors’ contributions

G. Stoll organized the project, set up the algorithms, participated in writing the software, set up the examples and wrote the article. E. Viara wrote the software and participated in setting up the algorithms. E. Barillot participated in discussions and corrected the manuscript. L. Calzone organized the project, set up the examples and wrote the article. All authors read and approved the final manuscript.

## Supplementary Material

Additional file 1 **Supplementary material.** Basic information on Markov process, abbreviations, definitions and algorithms. Click here for file

Additional file 2 **Model of the mammalian cell cycle with GINsim, BoolNet and MaBoSS.** The cell cycle presented in the “Examples” section has been modeled using three tools: GINsim, BoolNet, and MaBoSS. The results for each tool are presented: (1) GINsim provides steady state solutions and transition graphs for two different initial conditions: when CycD=0 and CycD=1. For the synchronous strategy, the transition graph can be visualized whereas for the asynchronous strategy, it is not easy to read or use; BoolNet constructs two graphical representations of the trajectories based on synchronous update strategy, for the case of CycD=0 (steady state) and CycD=1 (cycle); (3) MaBoSS estimates indecomposable stationary distributions for the case of CycD=0 (one fixed point, not shown) and CycD=1 (distribution of probabilities of different network states), and time-dependent activities of the cyclins showing damped oscillations. All results are coherent but are presented differently with a different focus for each tool. Click here for file

## References

[B1] Kauffman S Homeostasis and differentiation in random genetic control networks Nature 1969 224 177 178 10.1038/224177a0 5343519

[B2] Thomas R Regulatory networks seen as asynchronous automata: a logical description J Theor Biol 1991 153 1 23 10.1016/S0022-5193(05)80350-9

[B3] Stoll G Rougemont J Naef F Few crucial links assure checkpoint efficiency in the yeast cell-cycle network Bioinformatics 2006 22 20 2539 2546 10.1093/bioinformatics/btl432 16895923

[B4] Stoll G Bischofberger M Rougemont J Naef F Stabilizing patterning in the Drosophila segment polarity network by selecting models in silico Biosystems 2010 102 3 10 10.1016/j.biosystems.2010.07.014 20655356

[B5] Garg A Mohanram K Di Cara A De Micheli G Xenarios I Modeling stochasticity and robustness in gene regulatory networks Bioinformatics 2009 25 12 i101—i109 19477975 10.1093/bioinformatics/btp214 PMC2687968

[B6] Fauré A Chaouiya C Thieffry D Naldi A Dynamical analysis of a generic Boolean model for the control of the mammalian cell cycle Bioinformatics 2006 22 14 e124—e131 16873462 10.1093/bioinformatics/btl210

[B7] Shmulevich I Dougherty E Kim S Zhang W Probabilistic Boolean networks: a rule-based uncertainty model for gene regulatory networks Bioinformatics 2002 18 2 261 10.1093/bioinformatics/18.2.261 11847074

[B8] Gonzalez A Naldi A Sanchez L Thieffry D Chaouiya C GINsim: a software suite for the qualitative modelling, simulation and analysis of regulatory networks Biosystems 2006 84 2 91 100 10.1016/j.biosystems.2005.10.003 16434137

[B9] Wunderlich Z DePace A Modeling transcriptional networks in Drosophila development at multiple scales Curr Opin Genet Dev 2011 21 6 711 718 10.1016/j.gde.2011.07.005 21889888

[B10] MacArthur B Ma’ayan A Lemischka I Systems biology of stem cell fate and cellular reprogramming Nat Rev Mol Cell Biol 2009 10 10 672 681 19738627 10.1038/nrm2766 PMC2928569

[B11] Saez-Rodriguez J Alexopoulos L Zhang M Morris M Lauffenburger D Sorger P Comparing signaling networks between normal and transformed hepatocytes using discrete logical models Cancer Res 2011 71 16 5400 10.1158/0008-5472.CAN-10-4453 21742771 PMC3207250

[B12] Siebert H Bockmayr A Temporal constraints in the logical analysis of regulatory networks Theor Comput Sci 2008 391 3 258 275 10.1016/j.tcs.2007.11.010

[B13] Öktem H Pearson R Yli-Harja O Nicorici D Egiazarian K Astola J A computational model for simulating continuous time Boolean networks Proceedings of IEEE International Workshop on Genomic Signal Processing and Statistics (GENSIPS’02) October 2002 NC, USA: Raleigh

[B14] Vahedi G Faryabi B Chamberland J Datta A Dougherty E Sampling-rate-dependent probabilistic Boolean networks J Theor Biol 2009 261 4 540 547 10.1016/j.jtbi.2009.08.026 19716832

[B15] Sevim V Gong X Socolar J Reliability of transcriptional cycles and the yeast cell-cycle oscillator PLoS Comput Biol 2010 6 7 e1000842 10.1371/journal.pcbi.1000842 20628620 PMC2900291

[B16] Teraguchi S Kumagai Y Vandenbon A Akira S Standley D Stochastic binary modeling of cells in continuous time as an alternative to biochemical reaction equations Phys Rev E 2011 062903 6 10.1103/PhysRevE.84.062903 22304139

[B17] Bauer A Jackson T Jiang Y Rohlf T Receptor cross-talk in angiogenesis: mapping environmental cues to cell phenotype using a stochastic, Boolean signaling network model J Theor Biol 2010 264 3 838 846 10.1016/j.jtbi.2010.03.025 20307549

[B18] Abou-Jaoudé W Ouattara D Kaufman M From structure to dynamics: frequency tuning in the p53-Mdm2 network: I. Logical approach J Theor Biol 2009 258 4 561 577 10.1016/j.jtbi.2009.02.005 19233211

[B19] Van Kampen N Stochastic Processes in Physics and Chemistry 2004 Amsterdam, Netherlands: Elsevier

[B20] Shiryaev A Probability, volume 95 of Graduate texts in mathematics 1996 Springer-Verlag: New York, USA

[B21] Chaves M Albert R Sontag E Robustness and fragility of Boolean models for genetic regulatory networks J Theor Biol 2005 235 3 431 449 10.1016/j.jtbi.2005.01.023 15882705

[B22] Chaouiya C Petri net modelling of biological networks Briefings in Bioinformatics 2007 8 4 210 219 10.1093/bib/bbm029 17626066

[B23] Young W Elcock E Monte Carlo studies of vacancy migration in binary ordered alloys: I Proceedings of the Physical Society 1966 89 735 10.1088/0370-1328/89/3/329

[B24] Gillespie D A general method for numerically simulating the stochastic time evolution of coupled chemical reactions J Comput Phys 1976 22 4 403 434 10.1016/0021-9991(76)90041-3

[B25] Klamt S Saez-Rodriguez J Gilles E Structural and functional analysis of cellular networks with CellNetAnalyzer BMC Syst Biol 2007 1 2 10.1186/1752-0509-1-2 17408509 PMC1847467

[B26] Müssel C Hopfensitz M Kestler H BoolNet–an R package for generation, reconstruction and analysis of Boolean networks Bioinformatics 2010 26 10 1378 1380 10.1093/bioinformatics/btq124 20378558

[B27] De Jong H Geiselmann J Hernandez C Page M Genetic Network Analyzer: qualitative simulation of genetic regulatory networks Bioinformatics 2003 19 3 336 344 10.1093/bioinformatics/btf851 12584118

[B28] Di Cara A Garg A De Micheli G Xenarios I Mendoza L Dynamic simulation of regulatory networks using SQUAD BMC Bioinf 2007 8 462 10.1186/1471-2105-8-462 PMC2238325 18039375

[B29] Novak B Tyson J A model for restriction point control of the mammalian cell cycle J Theor Biol 2004 230 4 563 579 10.1016/j.jtbi.2004.04.039 15363676

[B30] Sahin Ö Fröhlich H Löbke C Korf U Burmester S Majety M Mattern J Schupp I Chaouiya C Thieffry D Modeling ERBB receptor-regulated G1/S transition to find novel targets for de novo trastuzumab resistance BMC Syst Biol 2009 3 1 10.1186/1752-0509-3-1 19118495 PMC2652436

[B31] Schlatter R Schmich K Vizcarra I Scheurich P Sauter T Borner C Ederer M Merfort I Sawodny O ON/OFF and beyond-a boolean model of apoptosis PLoS Comput Biol 2009 5 12 e1000595 10.1371/journal.pcbi.1000595 20011108 PMC2781112

