# Supplementary material for: Continuous time boolean modeling for biological signaling: application of Gillespie algorithm
Source: BMC Syst Biol. 2012 Aug 29;6:116. doi: 10.1186/1752-0509-6-116 (PMC3517402; doi:10.1186/1752-0509-6-116)
Supplement: Additional file 1 — Supplementary material. Basic information on Markov process, abbreviations, definitions and algorithms. [file 1752-0509-6-116-S1.pdf]

Basic information on Markov process, abbreviations, definions, algorithms and estimates for Continuous time Boolean modeling for biological signaling: application of Gillespie algorithm

Gautier Stoll*[1,2,3], Eric Viara[4] , Emmanuel Barillot[1,2,3] Laurence Calzone[1,2,3]

[1]Institut Curie, 26 rue d'Ulm, Paris, F-75248 France
[2]INSERM, U900, Paris, F-75248 France
[3]Mines ParisTech, Fontainebleau, F-77300 France
[4]Sysra, Yerres, F-91330 France

Email: Gautier Stoll*- gautier.stoll@curie.fr; Eric Viara - viara@sysra.com; Emmanuel Barillot - emmanuel.barillot@curie.fr; Laurence Calzone - laurence.calzone@curie.fr;

*Corresponding author

1 Basic information on Markov Process

Formally, a random variable (and by extension a stochastic process) is a function from a probability space to a state space Σ. We will consider only the case when Σ finite *i.e.* $|\Sigma| = m < \infty$. This is true in particular when Σ is a network state space: $|\Sigma| = 2^n < \infty$, where n is the number of network nodes. Our work is based on two books:

- Stochastic Processes in Physics and Chemistry, 2004, NG Van Kampen, Elsevier, Amsterdam.
- Probability, 1996, AN Shiryaev, volume 95 of Graduate texts in mathematics, Springer-Verlag, New York.

We provide here the demonstration of every theorem in order to present the theory in a self-consistent manner. These demonstrations can also be obtained from more general textbooks of Markov processes.

1.1 Definitions

A stochastic process is a set of random variables $\{s(t), t \in I \subset \mathbb{R}\}$ defined on a probability space. Formally, $s(t)$ is an application $\Omega \to \Sigma$, where Ω is the set of elementary events in the probability space. The full

probabilistic model is defined by joint probability densities, *i.e.* $\mathbf{P}\left[\{s(t)=\mathbf{S}_t\}\right]$, for any set $\{\mathbf{S}_t \in \Sigma, t \in J \subset I\}$.

Because such a mathematical model can be very complicated, stochastic processes are often restricted to Markov processes: a Markov process is a stochastic process that has the Markov property, expressed in the following way: "conditional probabilities in the future, related to the present and the past, depend only on the present". This property can be translated as follows:

$$\begin{aligned}
&\mathbf{P}\left[s(t_i)=\mathbf{S}^{(i)}|s(t_1)=\mathbf{S}^{(1)}, s(t_2)=\mathbf{S}^{(2)},\ldots,s(t_{i-1})=\mathbf{S}^{(i-1)}\right]\\
&= \quad \mathbf{P}\left[s(t_i)=\mathbf{S}^{(i)}|s(t_{i-1})=\mathbf{S}^{(i-1)}\right]
\end{aligned} \tag{1}$$

For the case of discrete time, *i.e.* $I=\{t_1,t_2,\ldots\}\subset\mathbb{N}$, it can be shown that a Markov process is completely defined by its transition probabilities ($\mathbf{P}\left[s(t_i)=\mathbf{S}|s(t_{i-1})=\mathbf{S}'\right]$) and its initial condition ($\mathbf{P}\left[s(t_1)=\mathbf{S}\right]$). For the case of continuous time, this can be generalized. If I is an interval ($I=[t_m,t_M]$), it can be shown (see Shiryaev) that a Markov process is completely defined by the set of transition rates $\rho_{(\mathbf{S}\to\mathbf{S}')}$ and its initial condition $\mathbf{P}\left[s(t_m)=\mathbf{S}\right]$. In that case, instantaneous probabilities $\mathbf{P}\left[s(t)=\mathbf{S}\right]$ are solutions of a master equation:

$$\frac{d}{dt}\mathbf{P}\left[s(t)=\mathbf{S}\right]=\sum_{\mathbf{S}'}\left\{\rho_{(\mathbf{S}'\to\mathbf{S})}\,\mathbf{P}\left[s(t)=\mathbf{S}'\right]-\rho_{(\mathbf{S}\to\mathbf{S}')}\,\mathbf{P}\left[s(t)=\mathbf{S}\right]\right\} \tag{2}$$

Formally, the transition rates (or the transition probabilities in the case of discrete time) can depend explicitly on time. For now, we will consider time independent transition rates. It can be shown that, according to this equation, the sum of probabilities over the network state space is constant. Obviously, the master equation represents a set of linear equations. Because the network state space is finite, $\mathbf{P}\left[s(t)=\mathbf{S}\right]$ can be seen as a vector of real numbers, indexed in the network state space: $\Sigma=\left\{\mathbf{S}^{(\mu)},\mu=1,\ldots,2^n\right\}$, $\vec{\mathbf{P}}(t)\Big|_{\mu}\equiv\mathbf{P}\left[s(t)=\mathbf{S}^{(\mu)}\right]$. With this notation, the master equation becomes:

$$\frac{d}{dt}\vec{\mathbf{P}}(t)=M\vec{\mathbf{P}}(t) \tag{3}$$

with

$$M|_{\mu\nu}\equiv\rho_{(\mathbf{S}^{(\nu)}\to\mathbf{S}^{(\mu)})}-\sum_{\sigma}\rho_{(\mathbf{S}^{(\nu)}\to\mathbf{S}^{(\sigma)})}\delta_{\mu\nu} \tag{4}$$

M is called the transition matrix. The solution of the master equation can be written formally:

$$\vec{\mathbf{P}}(t)=\exp(Mt)\vec{\mathbf{P}}(0) \tag{5}$$

Solutions of the master equation provide not only the instantaneous probabilities, but also conditional probabilities:

$$\mathbf{P}\left[s(t) = \mathbf{S}^{(\mu)} | s(0) = \mathbf{S}^{(\nu)}\right] = \left[\exp(Mt)\vec{\mathbf{P}}(0)\right]_{\mu} \tag{6}$$

with the initial condition

$$\vec{\mathbf{P}}(0)\Big|_{\sigma} = \delta_{\nu\sigma} \tag{7}$$

From this continuous time Markov process, a discrete time Markov process can be constructed (called a jump process) by defining the transition probabilities in the following way:

$$\mathbf{P}\left[s(t_i) = \mathbf{S}' | s(t_{i-1}) = \mathbf{S}\right] = \rho_{(\mathbf{S}\to\mathbf{S}')} / \sum_{\mathbf{S}''} \rho_{(\mathbf{S}\to\mathbf{S}'')} \tag{8}$$

1.2 Stationary distributions of continuous time Markov process

We present here the characterization of stationary distributions. In particular, we show that the time average of any single trajectory produced by Kinetic Monte-Carlo converges to an indecomposable stationary distribution (for a given state $\mathbf{S}$, the time average of a single trajectory $\hat{\mathbf{S}}(t), t \in [0, T]$ is given by $\frac{1}{T}\int_0^T dt I_{\mathbf{S}}(t)$, with $I_{\mathbf{S}}(t) \equiv \delta_{\mathbf{S},\hat{\mathbf{S}}(t)}$).

We define the concept of indecomposable stationary distribution associated to a set of transition rates as follows: it is a stationary continuous time Markov process, associated to the set of transition rates, for which instantaneous probabilities cannot be expressed as the linear combination of two (different) instantaneous probabilities that are themselves associated to the same set of transition rates.

In addition, let $G(\Sigma, E)$ be the graph on the network state space Σ (the transition graph) that associates an edge to each non-zero transition rate, *i.e.* $e(\mathbf{S}, \mathbf{S}') \in E$ if $\rho_{\mathbf{S}\to\mathbf{S}'} > 0$. Consider the set of strongly connected components. Because reduction of these components to a single vertex produces an acyclic graph, there exists at least one strongly connected component that has no outgoing edges. Let $\mathcal{F}_G = \{\Phi_k(\phi_k, e_k), k = 1, ..., s\}$ be the set of these connected components with no outgoing edges. In addition, let us recall that the support of a probability distribution is the set of states with non-zero probabilities.

The characterization of stationary distributions will be done by showing the following statements:

1. The support of any stationary distribution is the union of elements of $\mathcal{F}_G$.

2. Two stationary distributions that have the same support in a $\Phi \in \mathcal{F}_G$ are identical. Therefore, indecomposable stationary distributions are associated with elements of $\mathcal{F}_G$.

3. Probabilities of an indecomposable stationary distribution can be computed by averaging on infinite time over instantaneous probability, with an initial condition having a support in a $\Phi \in \mathcal{F}_G$.

4. Given an indecomposable stationary distribution, the time average of any trajectory of this process converges to this stationary distribution.

Lemma 1. *Consider a continuous time Markov process $s(t)$ which is stationary. Let $G(\Sigma, E)$ be the graph associated with the transition rates (transition graph). Let $H(V, F) \subset G$ a sub-graph with no outgoing edges. Let ∂V be the set of nodes (or states) that have an edge to H. $\forall \mathbf{S} \in \partial V$, $\mathbf{P}[s(t) = \mathbf{S}] = 0$.*

Proof. Consider the master equation applied to the sum of probabilities on V. Using the definition of V and ∂V (recall that the Markov process is stationary),

$$\begin{aligned} 0 &= \sum_{\mathbf{S} \in V} \frac{d}{dt} \mathbf{P}[s(t) = \mathbf{S}] \\ &= \sum_{\mathbf{S} \in V, \mathbf{S}' \in (V \bigcup \partial V)} (\rho_{\mathbf{S}' \to \mathbf{S}} \mathbf{P}[s(t) = \mathbf{S}'] - \rho_{\mathbf{S} \to \mathbf{S}'} \mathbf{P}[s(t) = \mathbf{S}]) \\ &= \sum_{\mathbf{S} \in V, \mathbf{S}' \in \partial V} \rho_{\mathbf{S}' \to \mathbf{S}} \mathbf{P}[s(t) = \mathbf{S}'] \end{aligned} \tag{9}$$

By definition of V and ∂V, $\forall \mathbf{S}' \in \partial V$, $\exists \mathbf{S} \in V$ such that $\rho_{\mathbf{S}' \to \mathbf{S}}$ is non-zero; then the equation above implies that $\mathbf{P}[s(t) = \mathbf{S}'] = 0$ □

Theorem 1. *Consider a continuous time Markov process $s(t)$ which is stationary. Let $G(\Sigma, E)$ be the graph associated with the transition rates (transition graph). Let $\mathcal{F}_G = \{\Phi_k(\phi_k, e_k), k = 1, ..., s\}$ be the set of the connected components with no outgoing edges. The set $\{\mathbf{S}$ s. th. $\mathbf{P}[s(t) = \mathbf{S}] > 0\}$ is the union of some of the ϕ_k.*

Proof. If a state $\mathbf{S}$ has a zero instantaneous probability $\mathbf{P}[s(t) = \mathbf{S}]$, all states $\mathbf{S}'$ that have a connection $\mathbf{S}' \to \mathbf{S}$ in G have also a zero instantaneous probability. This can be easily checked by applying the master equation to $\mathbf{P}[s(t) = \mathbf{S}]$.

Consider all states that have a connection to one of the ϕ_k; they have zero instantaneous probabilities according to the previous lemma. Then, by applying iteratively the previous statement, all states that do not belong to one of the ϕ_k have zero instantaneous probabilities.

It remains to show that if a state that belongs to one of the ϕ_k has a non-zero instantaneous probability, all states in ϕ_k have non-zero probabilities. Suppose that this is not true, *i.e.* there exists $\mathbf{S}, \mathbf{S}' \in \phi_k$ such that $\mathbf{P}[s(t) = \mathbf{S}] = 0$ and $\mathbf{P}[s(t) = \mathbf{S}'] > 0$. By the definition of the notion of strongly connected

component, there exists a path in Φ_k from $\mathbf{S}'$ to $\mathbf{S}$. Then, the statement at the beginning can be applied iteratively (along the path), producing a contradiction. □

Corollary 1. *Consider a set of transition rates. Let $G(\Sigma, E)$ be the graph associated with the transition rates (transition graph). Let $\mathcal{F}_G = \{\Phi_k(\phi_k, e_k), k = 1, ..., s\}$ be the set of connected components with no outgoing edges. Any stationary continuous time Markov process with these transition rates that is indecomposable has a support in $\mathcal{F}_G$.*

Proof. Almost direct from the previous theorem. □

Theorem 2. *Consider two different stationary Markov processes that have the same transition rates and the same support (states with non-zero instantaneous probabilities). If both stationary distributions are indecomposable (i.e. associated to the same strongly connected component), they are identical.*

Proof. If M is the transition matrix, and $\vec{\mathbf{P}}$, $\vec{\tilde{\mathbf{P}}}$ are two stationary distributions, we have:

$$M\vec{\mathbf{P}} = M\vec{\tilde{\mathbf{P}}} = 0 \text{ with } \sum_\mu \mathbf{P}_\mu = \sum_\mu \tilde{\mathbf{P}}_\mu = 1 \tag{10}$$

Consider $\vec{\mathbf{P}}^{(\alpha)} = \alpha\vec{\mathbf{P}} + (1-\alpha)\vec{\tilde{\mathbf{P}}}$. For $\alpha \in [0,1]$, $\vec{\mathbf{P}}^{(\alpha)}$ is also a stationary distribution according to M ($M\vec{\mathbf{P}}^{(\alpha)} = 0$, all components are between 0 and 1 and their sum is equal to 1). If $\alpha \notin [0,1]$, $\vec{\mathbf{P}}^{(\alpha)}$ may not be a stationary distribution because some components may be negative (and other bigger than 1, because the sum of components remains equal to 1). Consider

$$\alpha_m = \max_\alpha \left\{ \alpha < 0 \text{ s. t. } \exists\mu \text{ with } \mathbf{P}^{(\alpha)}_\mu = 0 \right\} \tag{11}$$

α_m exists for the following argument. There is at least one μ for which $\mathbf{P}_\mu \neq \tilde{\mathbf{P}}_\mu$. Because the sum of the components is always equal to 1, there exists one ν such that $\mathbf{P}_\nu > \tilde{\mathbf{P}}_\nu$. In that case, $\mathbf{P}^{(\alpha)}_\nu$ is a linear function of α with a positive slope, and can be set to zero by a negative value of α. Because there is a finite number of such α, α_m exists.

By definition of α_m, $\vec{\mathbf{P}}^{(\alpha_m)}$ is a stationary distribution for M, and components of α_m are all positive except one (α_m is the maximum negative value that sets one component to zero, implying that other components remain non-negative). Therefore, the support of $\vec{\mathbf{P}}^{(\alpha_m)}$ is smaller than $\vec{\mathbf{P}}$ and $\vec{\tilde{\mathbf{P}}}$, which contradicts the previous theorem. □

Theorem 3. *Consider a continuous time Markov process $s(t)$ whose initial condition has its support in a strongly connected component with no outgoing edges ϕ. The infinite time average of instantaneous*

probabilities converges to the stationary distribution associated to the same transitions rates with support in ϕ (this theorem shows the existence of an indecomposable stationary distribution associated to ϕ).

Proof. Consider the finite time average of probabilities:

$$\mathbf{P}_T(\mathbf{S}) \equiv \frac{1}{T}\int_0^T dt\, \mathbf{P}\left[s(t)=\mathbf{S}\right] \tag{12}$$

Let M be the transition matrix, $\vec{\mathbf{P}(t)}$ and $\vec{\mathbf{P}_T}$ are equivalent to $\mathbf{P}\left[s(t)=\mathbf{S}\right]$ and $\mathbf{P}_T(\mathbf{S})$. By definition, the components $\vec{\mathbf{P}_T}$ are non-negative and their sum is equal to one. Applying M on $\vec{\mathbf{P}_T}$, we obtain:

$$M\vec{\mathbf{P}}_T = \frac{1}{T}\int_0^T dt\, \frac{d}{dt}\vec{\mathbf{P}}(t) = \frac{1}{T}\left[\vec{\mathbf{P}}(T) - \vec{\mathbf{P}}(0)\right] \tag{13}$$

Therefore, $\lim_{T\to\infty} M\vec{\mathbf{P}}_T = 0$, because component of $\vec{\mathbf{P}}(t)$ is bounded. Because the space of $\vec{\mathbf{P}}_T$ is compact and because components of $\vec{\mathbf{P}}_T$ are bounded, there exists a converging sub-sequence $\vec{\mathbf{P}}_{T_i}, i = 1, \ldots$. Therefore, $\vec{\tilde{\mathbf{P}}} \equiv \lim_{i\to\infty}\vec{\mathbf{P}}_{T_i}$ is a stationary distribution associated to M. By the choice of the initial condition, instantaneous probabilities are always zero for states outside of ϕ; therefore the support of $\vec{\tilde{\mathbf{P}}}$ is in ϕ. Because there exists only one such stationary distribution (previous theorem), each converging sub-sequence of $\vec{\mathbf{P}}_T$ has the same limit. Therefore, $\vec{\mathbf{P}}_T$ converges to the unique indecomposable stationary distribution with its support in ϕ. □

Theorem 4. *Let $s(t)$ be a continuous time Markov process whose initial condition has its support in a strongly connected component with no outgoing edges ϕ. The limit $t \to \infty$ of instantaneous probabilities converges to the indecomposable stationary distribution associated to ϕ.*

Proof. Let us restrict the state space Σ to the strongly connected component ϕ and define the master equation as $\frac{d}{dt}\vec{\mathbf{P}}(t) = M\vec{\mathbf{P}}(t)$. By the previous theorem, there exists only one $\vec{\mathbf{P}}^{(0)}$ such that $M\vec{\mathbf{P}}^{(0)} = 0$ with $\mathbf{P}_i^{(0)} \in]0,1[\ \forall i = 1,\ldots m$. In addition, it can be shown that any solution with such an initial condition $\mathbf{P}_i(0) \in [0,1]\ \forall i = 1,\ldots m$ and $\sum_i \mathbf{P}_i(0) = 1$ has the following property: $\mathbf{P}_i(t) \in]0,1[\ \forall i = 1,\ldots\ \forall t > 0$. For that, suppose the converse: because the solutions of the master equation solutions are continuous, consider the smallest $\tilde{t} > 0$ such that $\exists\tilde{\mathbf{S}}$ with $\mathbf{P}\left[s(\tilde{t}) = \tilde{\mathbf{S}}\right] = 0$. Therefore

$$\frac{d}{dt}\mathbf{P}\left[s(\tilde{t}) = \tilde{\mathbf{S}}\right] = \sum_{\mathbf{S}'}\rho_{\mathbf{S}'\to\tilde{\mathbf{S}}}\mathbf{P}\left[s(\tilde{t}) = \tilde{\mathbf{S}'}\right] \geq 0 \tag{14}$$

The case $\frac{d}{dt}\mathbf{P}\left[s(\tilde{t}) = \tilde{\mathbf{S}}\right] > 0$ is impossible, because before $\tilde{t}$, all instantaneous probabilities are non-negative by definition of $\tilde{t}$, and because the master equation solutions are continuous*. Therefore,

*notice that probabilities cannot be negative in a neighborhood of $t = 0$, because of the equation (14)

$\frac{d}{dt}\mathbf{P}\left[s(\tilde{t}) = \tilde{\mathbf{S}}\right] = 0$ at $t = \tilde{t}$ and all states that have a target to $\tilde{\mathbf{S}}$ have also a zero probability (equation 14). By applying this statement iteratively, because the system is restricted to a strongly connected component, all states have zero probability at time $\tilde{t}$, which is a contradiction. Therefore, for $t > 0$, all states have non-zero positive probability. Because the sum of probabilities is constantly equal to one, then $\mathbf{P}_i(t) \in]0, 1[\ \forall i = 1, \ldots\ \forall t > 0$.

Consider the spectral decomposition of M: $\left\{\lambda_i, \vec{v}^{(i)}\right\}$. $\vec{\mathbf{P}}^{(0)} = \vec{v}^{(i)}$ for $\lambda_i = 0$. Any solution has the form $\sum_i \beta_i \exp(t\lambda_i)\vec{v}^{(i)}$. If M is non-diagonalizable, one should multiply $\exp(t\lambda_i)$ by a polynomial in t. In order to have the property $\sum_i \mathbf{P}_i(t) = \text{constant}$, one should have $\sum_j v_j^{(i)} = 0$ for i such that $\lambda_i \neq 0$. Therefore, any solution with $\sum_i \mathbf{P}_i(t) = 1$ is the linear combination of $\vec{\mathbf{P}}^{(0)}$ and of other time varying solution(s). The constant coefficient in front of any time varying solutions can be set as small as possible, such that the initial conditions of probabilities are in $[0, 1]$. In that case, the property $\mathbf{P}_i(t) \in]0, 1[\ \forall i = 1, \ldots\ \forall t > 0$ implies that $\Re\lambda_i \leq 0\ \forall \lambda_i$.

It remains to show that an oscillatory solution is impossible ($\Re\lambda_i < 0\ \forall \lambda_i \neq 0$). Suppose the converse: let $\vec{\mathbf{P}} = \alpha\vec{\mathbf{P}}^{(0)} + \beta\vec{\mathbf{P}}^s(t)$ be a solution of the master equation, with $\vec{\mathbf{P}}^s(t)$ being an oscillatory solution. It is possible to tune α and β in order to have $\sum_i \mathbf{P}_i(t) = 1$ and $\mathbf{P}_i(t) \in]0, 1[\ \forall i = 1, \ldots\ \forall t > 0$. Because β can be constantly varied within an interval ($\sum_i \mathbf{P}_i^s) = 0$), it is possible to construct a β_M such that $\exists\ (j, \tilde{t} > 0)$ with $\mathbf{P}_j(\tilde{t}) = 0$ and $\mathbf{P}_i(t) \in [0, 1]\ \forall i = 1, \ldots\ \forall t > 0^{\dagger}$. But we have shown above that this is impossible. Therefore, $\Re\lambda_i < 0$ for $\lambda_i \neq 0$ and any time varying solution converges to the stationary solution $\vec{\mathbf{P}}^{(0)}$. □

Corollary 2. *For a continuous time Markov process to a finite state space, the limit $t \to \infty$ of instantaneous probabilities converges to a stationary distribution.*

Proof. As the previous theorem, consider $\frac{d}{dt}\vec{\mathbf{P}}(t) = M\vec{\mathbf{P}}(t)$. Consider the spectrum of M, *i.e.* $\left\{\lambda_i, \vec{v}^{(i)}\right\}$. Because any solution has $\sum_i \mathbf{P}_i(t) = \text{cst}$, $\sum_j v_j^{(i)} = 0$ for i such that $\lambda_i \neq 0$. With identical arguments as in the previous theorem, the fact that $\mathbf{P}_i(t) \in [0, 1]\ \forall i = 1, \ldots\ \forall t > 0$ implies that $\Re\lambda_i \leq 0\ \forall \lambda_i$. Consider $\vec{\mathbf{P}} = \alpha\vec{\mathbf{P}}^{(0)} + \beta\vec{\mathbf{P}}^s(t)$ with $\vec{\mathbf{P}}^s(t)$ an oscillatory solution. As for the theorem above, β and α can be tuned in order to have $\mathbf{P}_j(\tilde{t}) = 0$ for a given j and a given $\tilde{t}$. Again, all states that have a non-zero transition rate to state j have also zero probability at time $\tilde{t}$. By extension, the smallest sub-graph $H \subset G(\Sigma, E)$, containing the state j and having no incoming edges, has nodes with zero probability at time $\tilde{t}$. Because this set has no incoming edges, the probability of its nodes is zero for $t > \tilde{t}$ (and by extension for $t > 0$ because of

†the fact that an oscillatory solution is a linear combination of cosine and sine functions is crucial. This $\tilde{t}$ corresponds to a local minimum of a cos or a sin, which is also a global minimum. This argument does not work for damped oscillatory solutions: in that case, the increase of the coefficient in front of the damped oscillating solution will be stopped because the initial condition will have negative probabilities

uniqueness of solutions for any system of linear differential equations). Applying this argument to another state outside H, we conclude that $\vec{\mathbf{P}}^s(t)$ is zero everywhere. Therefore, $\Re\lambda_i < 0$ if $\lambda_i \neq 0$ and any time varying solution converges to a stationary one. □

Theorem 5. *Consider a continuous time Markov process applied on a discrete state space Σ. The time average along a single trajectory (produced by Kinetic Monte-Carlo for example) converges to a stationary distribution.*

Proof. We can first restrict the continuous time Markov process to a stationary Markov process in a single strongly connected component with no outgoing edges: there is a finite time τ after which the trajectory belongs to a strongly connected component with no outgoing edges; for $t > \tau$, the trajectory also belongs to the stationary Markov process associated with this strongly connected component with no outgoing edges. If the time average starting at τ converges, then the time average starting at any time converges to the same value.

For that, we apply definitions 1 & 2 and theorem 3 in chapter V, §3 in Shiryaev. Formally, the set of trajectories represents the set of elementary events $\omega \in \Omega$, with the right definition of the probability measure $\mathbf{P}$ on a given σ-algebra $\mathcal{F}$. The stationary sequence is given by instantaneous probabilities $\mathbf{P}\left[s(t_i) = \mathbf{S}\right]$ defined on equidistant discrete time $t_i = v * i, i = 1, \ldots,$. Note that stationarity of continuous time Markov process and definition of t_i imply that the discrete process is stationary and Markovian. Formally, a trajectory ω is a function $\mathbb{R} \to \Sigma, t \mapsto \omega_t$ and the stationary sequence is a set of random variables $\mathbb{N} \times \Omega \to \Sigma, (\omega, i) \mapsto \omega_{t_i}$. If we translate the definition 1 in our formalism, an invariant set $A \in \mathcal{F}$ is such that there exists $B = B_1 \times B_2 \times \ldots$ with $B_i \subset \Sigma$ such that, for all $n \geq 1$,

$$A = \left\{\omega \text{ s. th. } \left(\omega_{t_n}, \omega_{t_{n+1}}, \ldots\right) \in B\right\} \tag{15}$$

If $B = \Sigma \times \Sigma \times \ldots$, then $A = \Omega$ and $\mathbf{P}(A) = 1$. Consider the biggest set B that is "smaller" than $\Sigma \times \Sigma \times \ldots$. It consists of removing one element in one of the B_i. With no loss of generality, let us consider that $B_1 = \Sigma \backslash \{\mathbf{S}\}$. In that case:

$$A = \{\omega \text{ s. th. } \omega_{t_n} \neq \mathbf{S}\ \forall n \geq 1\} \tag{16}$$

Using Markov property:

$$\begin{aligned}\mathbf{P}(A) &= \mathbf{P}\left[s(t_1) \neq \mathbf{S}, s(t_2) \neq \mathbf{S}, \ldots\right] \\ &= \lim_{n\to\infty} \sum_{\mathbf{S}^{(1)}\ldots\mathbf{S}^{(n)}\neq\mathbf{S}} \mathbf{P}\left[s(t_1) = \mathbf{S}^{(1)}\right] \times \\ &\quad \times\mathbf{P}\left[s(t_2) = \mathbf{S}^{(2)}|s(t_1) = \mathbf{S}^{(1)}\right] \ldots \mathbf{P}\left[s(t_n) = \mathbf{S}^{(n)}|s(t_{n-1}) = \mathbf{S}^{(n-1)}\right]\end{aligned} \tag{17}$$

With theorem 4, we know that any solution of a master equation has non-zero probabilities (except for the initial condition). Because transition probabilities are computed from solutions of the master equation:

$$\sum_{\mathbf{S}'\neq\mathbf{S}} \mathbf{P}\left[s(t_1) = \mathbf{S}'|s(t_1) = \mathbf{S}''\right] \leq k < 1 \tag{18}$$

and because $\mathbf{P}\left[s(t_1) = \mathbf{S}|s(t_1) = \mathbf{S}''\right]$ is bigger than zero. k can be taken as independent of $\mathbf{S}''$, because there is a finite number of possible $\mathbf{S}''$. Therefore:

$$\mathbf{P}(A) \leq \lim_{n\to\infty} \sum_{\mathbf{S}^{(1)}\neq\mathbf{S}} \mathbf{P}\left[s(t_1) = \mathbf{S}^{(1)}\right] k^{n-1} = 0 \tag{19}$$

If A has zero probability, any sub-set has also zero probability. Therefore, the stationary sequence is ergodic (definition 2 in Shiryaev). Applying the ergodic theorem (theorem 3 in Shiryaev), the time average of the stationary sequence converges to an instantaneous probability distribution (which is the stationary distribution). If any discrete average converges to the same distribution, continuous time average converges also to the stationary distribution.

Remark: the fact that any solution of the master equation has non-zero probability (and that the state space Σ is finite) is enough to demonstrate ergodicity of the discrete Markov process (each transition probability is non-zero). But the definition of an ergodic Markov process does not obviously imply that the time average of a single elementary event converges to a stationary distribution. Because this fact is often not clearly demonstrated, we prefer to present a proof that uses the general definition of ergodicity. □

1.3 Oscillating solutions of the master equation

A complete analysis of the oscillatory behavior of a Markov process, given the initial condition and transition rates, is beyond the scope of the present work. Indeed, some general considerations can be stated. It has been shown above (proof of theorem 4) that any solution of the master equation is a linear

combination of a constant, exponential decays and damped exponential decays:

$$\mathbf{P}\left[s(t)=\mathbf{S}\right] = K(\mathbf{S}) + D(\mathbf{S},t) + F(\mathbf{S},t) \tag{20}$$

with

$$\begin{aligned} D(\mathbf{S},t) &= \sum_i d_i(\mathbf{S}) p_i(t) \exp(-\lambda_i t),\ \lambda_i > 0,\ p_i \text{ polynomial} \\ F(\mathbf{S},t) &= \sum_i f_i(\mathbf{S}) q_i(t) \exp(-\eta_i t) \cos(\omega_i t - \phi_i),\ \eta_i > 0,\ (\omega_i, \phi_i) \neq 0,\ q_i \text{ polynomial} \end{aligned} \tag{21}$$

where $K(\mathbf{S})$ is the stationary distribution towards which the process converges. It can be noticed that K, λ_i, η_i and ω_i depend only on the transition rates (or on the transition matrix).

Let us define formally a *damped oscillatory* Markov process: it is a process whose instantaneous probabilities have an infinite number of extrema, at least for one state. According to the decomposition of equation 20, the initial condition can be modified in order to lower the value of $|D(\mathbf{S},t)|$ and to increase $|F(\mathbf{S},t)|$ in order to have a damped oscillatory process as defined above; but this is only possible if η_i and ω_i exist. This can be reformulated in this simple theorem:

Theorem 6. *Consider a set of transition rates. It is possible to construct a damped oscillatory Markov process with these transition rates if and only if the transition matrix has at least one non-real eigenvalue.*

We provide some theorems about the existence of non-real eigenvalues:

Theorem 7. *A transition matrix, whose transition graph has no cycle, has only real eigenvalues.*

Proof. Consider the master equation:

$$\frac{d}{dt}\mathbf{P}\left[s(t)=\mathbf{S}\right] = \sum_{\mathbf{S}'} \left\{ \rho_{(\mathbf{S}' \to \mathbf{S})}\ \mathbf{P}\left[s(t)=\mathbf{S}'\right] - \rho_{(\mathbf{S} \to \mathbf{S}')}\ \mathbf{P}\left[s(t)=\mathbf{S}\right] \right\} \tag{22}$$

This equation can be rewritten:

$$\frac{d}{dt}\mathbf{P}\left[s(t)=\mathbf{S}\right] + \left(\sum_{\mathbf{S}'} \rho_{(\mathbf{S} \to \mathbf{S}')} \right) \mathbf{P}\left[s(t)=\mathbf{S}\right] = \sum_{\mathbf{S}'} \rho_{(\mathbf{S}' \to \mathbf{S})}\ \mathbf{P}\left[s(t)=\mathbf{S}'\right] \tag{23}$$

Or

$$\frac{d}{dt}\mathbf{P}\left[s(t)=\mathbf{S}\right] + K\mathbf{P}\left[s(t)=\mathbf{S}\right] = F(t) \tag{24}$$

Therefore,

$$\mathbf{P}\left[s(t)=\mathbf{S}\right] = e^{-Kt} p_0 + \int_0^t F(s) e^{K(s-t)} ds \tag{25}$$

where $F(t)$ depends only on instantaneous probabilities of upstream states (in the transition graph). Because the transition graph has no cycle, probabilities of upstream states do not depend on $\mathbf{P}\left[s(t)=\mathbf{S}\right]$. Therefore, every $\mathbf{P}\left[s(t)=\mathbf{S}\right]$ can be obtained iteratively by computing the left-hand side of equation 25, starting at states that have no incoming edges in the transition graph (and with specified initial conditions). Because this iterative procedure consists of integrating exponential functions, it will never produce an oscillatory function (sine or cosine). Therefore, the transition matrix has only real eigenvalues. □

Theorem 8. *Consider a transition matrix ($m \times m$), whose transition graph is a unique cycle, with identical transition rates. If the matrix dimension is bigger than 2×2, the matrix has at least one non-real eigenvalue.*

Proof. If the states are ordered along the cycle, the transition matrix (equation 4) becomes

$$\begin{aligned} M|_{\mu,\nu} &= \delta_{\mu,\nu}(-\rho) + \delta_{\mu,\nu+1}\rho \ \text{ for } \nu < m \\ M|_{\mu,m} &= \delta_{\mu,m}(-\rho) + \delta_{\mu,1}\rho \end{aligned} \tag{26}$$

where ρ is the transition rate.

The characteristic polynomial of M is

$$p_M(\lambda) = (\lambda+\rho)^m - \rho^m \tag{27}$$

This last equation can be easily obtained by applying the definition of the determinant: $\det(M) = \sum_\sigma \Pi_i \text{sgn}(\sigma) M_{i\sigma(i)})$. Therefore, the eigenvalues of M are

$$\lambda_k = \rho e^{i2\pi k/m} - 1 \text{ with } k = 1 \ldots m \tag{28}$$

Therefore, if $m > 2$, there is at least one λ_k that is non-real. □

Corollary 3. *Consider a graph with at least one cycle. There exists a set of transition rates associated with this graph, whose transition matrix has at least one non-real eigenvalue.*

Proof. Consider a transition matrix M_0 that has identical transition rates associated with the cycle of the transition graph, and all other transition rates set to zero. According to the previous theorem, M_0 has one non-zero eigenvalue and therefore has damped oscillatory solution(s). Consider M_p, a perturbation of M_0, that consists of adding small transition rates associated with other links in the graph. Because any solution of the master equation is analytic in transition rates (matrix exponential is an analytic function), a small

perturbation of a damped oscillatory solution will remain qualitatively the same. Therefore M_p has also a damped oscillatory behavior if the new transition rates are small enough. Therefore, M_p has at least one non-real eigenvalue. □

Notice that the converse of this corollary is not true. It is possible to construct a parameter-dependent transition matrix where a continuous variation of transition rates transform non-real eigenvalue(s) to real one(s), which can be considered as a bifurcation.

2 Abbreviations

BKMC: Boolean Kinetic Monte-Carlo

AT: Asynchronous transition

ODEs: Ordinary Differential Equations

MaBoSS: Markov Boolean Stochastic Simulator

3 Definitions

Asynchronous transition of node i: Boolean transition $\mathbf{S} \to \mathbf{S}'$ such that $S'_i = B_i(\mathbf{S})$ and $S'_j = S_j, j \neq i$.

Boolean Kinetic Monte-Carlo: kinetic Monte-Carlo algorithm (or Gillespie algorithm) applied to continuous time Markov process applied on a network state space.

Cycle: loop in the transition graph (in Σ).

Cyclic stationary distribution of a stationary distribution: probability distribution such that states with non-zero probability have only one possible transition (to another state with non-zero probability).

Damped oscillatory Markov process: continuous time process that has at least one instantaneous probability with an infinite number of extrema.

Entropy at a given time window τ, $H(\tau)$: Shannon entropy over network state probability on a time window:

$$H(\tau) \equiv -\sum_{\mathbf{S}} \log_2 \left(\mathbf{P}\left[s(\tau) = \mathbf{S}\right]\right) \mathbf{P}\left[s(\tau) = \mathbf{S}\right]$$

Fixed point of a stationary distribution: probability distribution having one state with probability one.

Hamming distance between two network states $\mathbf{S}$ and $\mathbf{S}'$, $HD(\mathbf{S}, \mathbf{S}')$: number of different node states between $\mathbf{S}$ and $\mathbf{S}'$:

$$HD(\mathbf{S}, \mathbf{S}') \equiv \sum_i (1 - \delta_{\mathbf{S}_i, \mathbf{S}'_i})$$

Hamming distance distribution of a Markov process, given a reference state $\mathbf{S}_{\text{ref}}$, $\mathbf{P}(HD,t)$: probability distribution of Hamming distance from the reference state:

$$\mathbf{P}(HD,t) \equiv \sum_{\mathbf{S}} \mathbf{P}\left[s(t)=\mathbf{S}\right] \delta_{HD,HD(\mathbf{S},\mathbf{S}_{\text{ref}})}$$

Indecomposable stationary distribution: stationary distribution that cannot be expressed as a linear combination of (different) stationary distributions.

Inputs Nodes: nodes on which the initial condition is fixed.

Instantaneous probabilities (first order probabilities), $\mathbf{P}\left[s(t)=\mathbf{S}\right]$: for a stochastic process, probability distribution of a single random variable; in other words, probability distribution at a given time.

Internal Nodes: nodes that are not considered for computing probability distributions, entropies and transition entropies. But these internal nodes are used for generating time trajectories through BKMC algorithm.

Jump process associated of a continuous time Markov process: discrete time Markov process with the following transition probabilities:

$$\mathbf{P}_{\mathbf{S}\to\mathbf{S}'} \equiv \frac{\rho_{\mathbf{S}\to\mathbf{S}'}}{\sum_{\mathbf{S}''} \rho_{\mathbf{S}\to\mathbf{S}''}}$$

Kinetic Monte-Carlo (or *Gillespie algorithm*): algorithm for generating stochastic trajectories of a continuous time Markov process, given the set of transition rates. *Logic* of node i, $B_i(\mathbf{S})$: in asynchronous Boolean Dynamics, a Boolean function from state $\mathbf{S} \in \Sigma$ to node state $S_i \in \{0,1\}$.

Markov process: stochastic process having the Markov property: "conditional probabilities in the future, related to the present and the past, depend only on the present".

Master equation: differential equation for computing instantaneous probabilities from transition rates:

$$\frac{d}{dt}\mathbf{P}\left[s(t)=\mathbf{S}\right] = \sum_{\mathbf{S}'} \left\{ \rho_{(\mathbf{S}'\to\mathbf{S})}\, \mathbf{P}\left[s(t)=\mathbf{S}'\right] - \rho_{(\mathbf{S}\to\mathbf{S}')}\, \mathbf{P}\left[s(t)=\mathbf{S}\right] \right\}$$

Network state, $\mathbf{S}$: for a given set of nodes, vector of node states.

Network states probability on a time window over time interval Δt, $\mathbf{P}\left[s(\tau)=\mathbf{S}\right]$: instantaneous probabilities that are averaged over time interval:

$$\mathbf{P}\left[s(\tau)=\mathbf{S}\right] \equiv \frac{1}{\Delta t}\int_{\tau\Delta t}^{(\tau+1)\Delta t} dt\, \mathbf{P}\left[s(t)=\mathbf{S}\right]$$

Network state space, Σ: set of all possible network states $\mathbf{S}$ for a given set of nodes. The size is $2^{\#\text{nodes}}$.

Output nodes: nodes that are not internal.

Set of *realizations* or *stochastic trajectories* of a given stochastic process: set of time trajectories in network state space, $\hat{\mathbf{S}}(t) \in \Sigma, t \in I \subset \mathbb{R}$, that corresponds to the set of elementary events of the stochastic process.

Reference Nodes: nodes for which there is a reference state; the Hamming distance is computed considering only these nodes.

Similarity coefficient, $D(s_0^{(i)}, s_0^{(j)}) \in [0,1]$ between two stationary distribution estimates $s_0^{(i)}$ and $s_0^{(j)}$: real number quantifying how close these two estimates are.

State of the node i, S_i: Boolean value (0 or 1) associated to node indexed by i.

Stationary stochastic process: stochastic process with constant joint probabilities respective to global time shift. The consequence is that instantaneous probabilities are time independent).

Stationary distribution of a Markov process: instantaneous probabilities associated to a (new) stationary Markov process having the same transition probabilities/rates.

Stochastic process, $(s : t \in I \subset \mathbb{R} \mapsto s(t))$: set of random variables indexed by an real/integer number (called "time"), over the same probability space. Notice that, within this definition, a stochastic process is defined from a probability space to a state space. If time is an integer number, the stochastic process is called discrete. If time is a real number, stochastic process is called continuous.

Time independent Markov process: Markov process with time independent transition probabilities/rates.

Transition Entropy of state $\mathbf{S}$, $TH(\mathbf{S})$: Shannon entropy over probability distribution of transitions from $\mathbf{S}$:

$$TH(\mathbf{S}) \equiv -\sum_{\mathbf{S}'} \log_2(\mathbf{P}_{\mathbf{S}\to\mathbf{S}'})\mathbf{P}_{\mathbf{S}\to\mathbf{S}'}$$

(by convention, $TH(\mathbf{S}) = 0$ if there is no transition from $\mathbf{S}$), with

$$\mathbf{P}_{\mathbf{S}\to\mathbf{S}'} \equiv \frac{\rho_{\mathbf{S}\to\mathbf{S}'}}{\sum_{\mathbf{S}''} \rho_{\mathbf{S}\to\mathbf{S}''}}$$

Transition Entropy with internal nodes of state $\mathbf{S}$:

- If the only possible transitions from state $\mathbf{S}$ consist of flipping an internal node, the transition entropy is zero.
- If the possible transitions consist of flipping internal or/and output nodes, only the output nodes will be considered for computing $\mathbf{P}_{\mathbf{S}\to\mathbf{S}'}$.

Transition Entropy on a time window, $TH(\tau)$: transition entropy over probability distributions of

transition from $\mathbf{S}$:

$$TH(\tau) \equiv \sum_{\mathbf{S}} \mathbf{P}\left[s(\tau) = \mathbf{S}\right] TH(\mathbf{S})$$

Transition graph of a time independent Markov process: graph in Σ, with an edge between $\mathbf{S}$ and $\mathbf{S}'$ when $\rho_{\mathbf{S}\to\mathbf{S}'} > 0$ (or $\mathbf{P}\left[s(t_i) = \mathbf{S}|s(t_{i-1}) = \mathbf{S}'\right] > 0$ if time is discrete).

Transition probabilities, $\mathbf{P}\left[s(t) = \mathbf{S}|s(t-1) = \mathbf{S}'\right]$: for discrete time Markov process, conditional probability distributions at a given time, given the state at previous time.

Transitions rates, $\rho_{\mathbf{S}\to\mathbf{S}'}$ (≥ 0): basic elements for constructing a continuous time Markov process, similar to transition probabilities for a discrete time Markov process.

4 Algorithms and estimates

Cluster of (estimated) stationary distributions given a similarity threshold α:

$$\mathcal{C} = \{s_0 |\ \exists s_0' \in \mathcal{C} \text{ s. t. } D(s_0, s_0') \geq \alpha\}$$

Cluster distribution estimate, given a cluster:

$$\mathbf{P}\left[s_{\mathcal{C}} = \mathbf{S}\right] = \frac{1}{|\mathcal{C}|} \sum_{s \in \mathcal{C}} \mathbf{P}\left[s = \mathbf{S}\right]$$

The error on these probabilities can be computed by

$$\text{Err}\left(\mathbf{P}\left[s_{\mathcal{C}} = \mathbf{S}\right]\right) = \sqrt{\text{Var}(\mathbf{P}\left[s = \mathbf{S}\right], s \in \mathcal{C})/|\mathcal{C}|}$$

Entropy on a time window τ from network state probabilities:

$$\hat{H}(\tau) = -\sum_{\mathbf{S}} \log_2\left(\hat{\mathbf{P}}\left[s(\tau) = \mathbf{S}\right]\right) \hat{\mathbf{P}}\left[s(\tau) = \mathbf{S}\right]$$

Hamming distance distribution on a time window τ from network state probabilities, given a reference state $\mathbf{S}_{\text{ref}}$:

$$\hat{\mathbf{P}}(HD, \tau) = \sum_{\mathbf{S}} \hat{\mathbf{P}}\left[s(\tau) = \mathbf{S}\right] \delta_{HD, HD(\mathbf{S}, \mathbf{S}_{\text{ref}})}$$

Kinetic Monte-Carlo (or *Gillespie algorithm*), given $\mathbf{S}$ and two uniform random numbers $u, u' \in [0, 1[$:

1. Compute the total rate of possible transitions for leaving state $\mathbf{S}$, *i.e.* $\rho_{\text{tot}} \equiv \sum_{\mathbf{S}'} \rho_{(\mathbf{S}\to\mathbf{S}')}$.

2. Compute the time of the transition: $\delta t \equiv -\log(u)/\rho_{\text{tot}}$

3. Order the possible new states $\mathbf{S}'^{(j)}, j = 1 \ldots$ and their respective transition rates $\rho^{(j)} = \rho_{(\mathbf{S}\to\mathbf{S}'^{(\mathbf{j})})}$.

4. Compute the new state $\mathbf{S}'^{(k)}$ such that $\sum_{j=0}^{k-1} \rho_j < (u' \rho_{\text{tot}}) \leq \sum_{j=0}^{k} \rho_j$ (by convention, $\rho^{(0)} = 0$).

Network states probability on a time window from a set of trajectories:

1. **Estimate for one trajectory.** For each trajectory j, compute the time for which the system is in state $\mathbf{S}$, in the window $[\tau \Delta t, (\tau+1)\Delta t]$. Divide this time by Δt. Obtain an estimate of $\mathbf{P}[s(\tau) = \mathbf{S}]$ for trajectory j, *i.e.* $\hat{\mathbf{P}}_j [s(\tau) = \mathbf{S}]$.

2. **Estimate for a set of trajectories.** Compute the average over j of all $\hat{\mathbf{P}}_j [s(\tau) = \mathbf{S}]$ to obtain $\hat{\mathbf{P}}[s(\tau) = \mathbf{S}]$. Compute the error of this average ($\sqrt{\text{Var}(\hat{\mathbf{P}}[s(\tau) = \mathbf{S}])/\# \text{ trajectories}}$).

Similarity coefficient between two stationary distributions estimates s_0, s_0':

$$D(s_0, s_0') = \left(\sum_{\mathbf{S} \in \text{supp}(s_0, s_0')} \hat{\mathbf{P}}[s_0 = \mathbf{S}] \right) \left(\sum_{\mathbf{S}' \in \text{supp}(s_0, s_0')} \hat{\mathbf{P}}[s_0' = \mathbf{S}'] \right)$$

where

$$\text{supp}(s_0, s_0') \equiv \left\{ \mathbf{S} | \ \hat{\mathbf{P}}[s_0 = \mathbf{S}] \, \hat{\mathbf{P}}[s_0' = \mathbf{S}] > 0 \right\}$$

Stationary distribution estimate from a single trajectory $\hat{\mathbf{S}}(t), t \in [0, T]$:

$$\hat{\mathbf{P}}[s_0 = \mathbf{S}] = \frac{1}{T} \int_0^T dt I_{\mathbf{S}}(t)$$

where $I_{\mathbf{S}}(t) \equiv \delta_{\mathbf{S}, \hat{\mathbf{S}}(t)}$

Transition Entropy on a time window τ from network state probabilities:

1. **Estimate for one trajectory.** For each trajectory j, compute the set Φ of visited states $\mathbf{S}$ in the time window $[\tau \Delta t, (\tau+1)\Delta t]$ and their respective duration $\mu_{\mathbf{S}}$). The estimated transition entropy is

$$T\hat{H}(\tau)_j = \sum_{\mathbf{S} \in \Phi} TH(\mathbf{S}) \frac{\mu_{\mathbf{S}}}{\Delta t}$$

2. **Estimate for a set of trajectories.** Compute the average over j of all $T\hat{H}(\tau)_j$ to obtain $T\hat{H}(\tau)$. Compute the error of that average ($\sqrt{\text{Var}(T\hat{H}(\tau))/\# \text{ trajectories}}$).
