# Supplementary material for: Continuous time boolean modeling for biological signaling: application of Gillespie algorithm
Source: BMC Syst Biol. 2012 Aug 29;6:116. doi: 10.1186/1752-0509-6-116 (PMC3517402; doi:10.1186/1752-0509-6-116)

Model of the mammalian cell cycle with 3 different tools

Original publication:
A.Fauré, A.Naldi, C.Chaouiya, D.Thieffy . Dynamical analysis of a generic Boolean model for the control of the mammalian cell cycle. Bioinformatics (2006); 22(14): 124-131

(1) GINsim results

Two asymptotic behaviors:

- With CycD = 0: one stable steady state (G1 arrest : Rb=1, p27=1, Cdh1=1)
- With CycD = 1: unique multi-cycle attractor

For CycD = 1, the state transition graphs according to two different updating strategies: synchronous and asynchronous are shown:

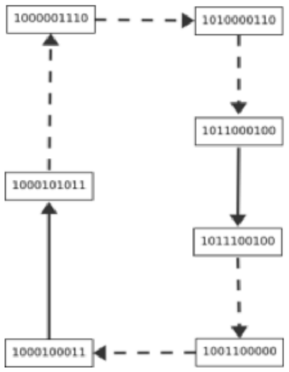

Synchronous strategy

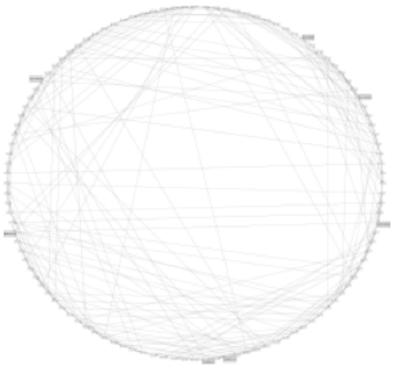

Asynchronous strategy

(2) BoolNet results

Two asymptotic behaviors are observed according to the initial state of CycD
(Note that BoolNet provides computations with synchronous update strategy)

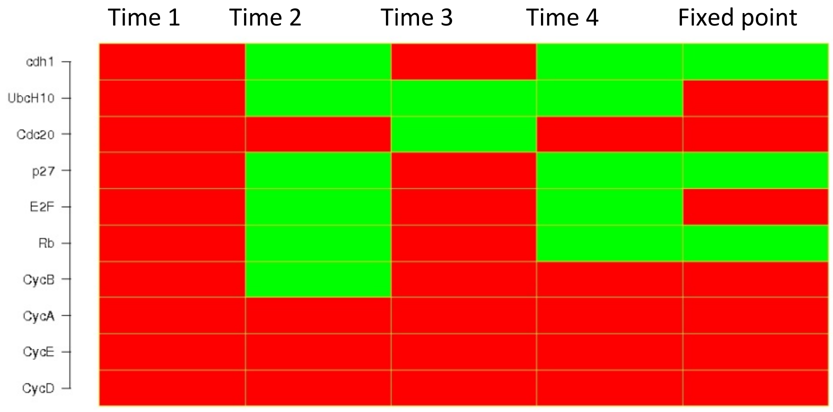

CycD = 0→ fixed point (G1 arrest : Rb p27 Cdh1)

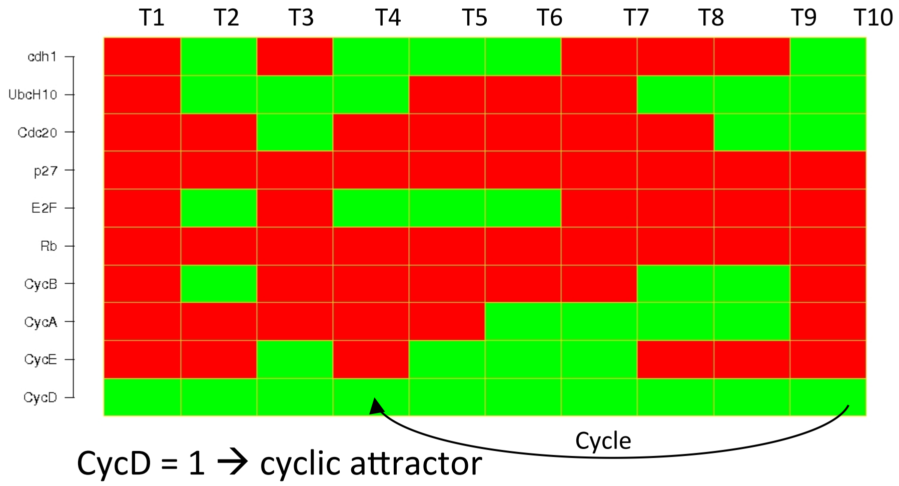

CycD = 1 → cyclic attractor

(3) MaBoSS results

Two indecomposable stationary distributions (clusters):

- One fixed point: Rb = 1, CDH1 = 1, p27 =1 (not shown)
- One multi-cyclic attractor

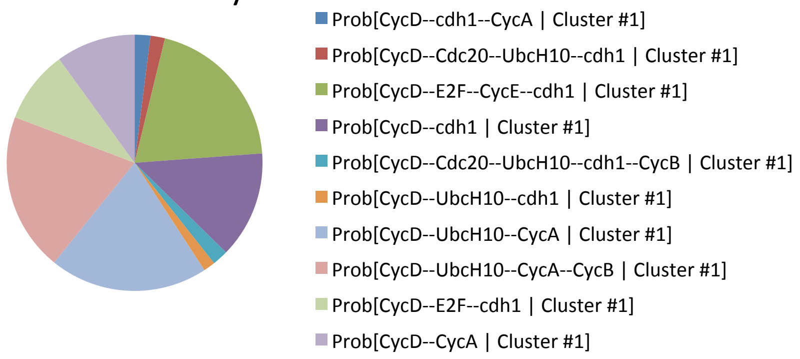

Time dependent activity of cyclins (initial condition: CycD=1)

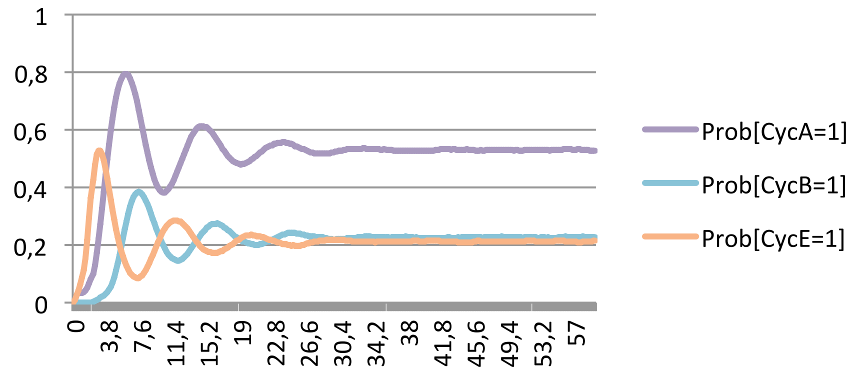

Supplement: Additional file 2 — Model of the mammalian cell cycle with GINsim, BoolNet and MaBoSS. The cell cycle presented in the “Examples” section has been modeled using three tools: GINsim, BoolNet, and MaBoSS. The results for each tool are presented: (1) GINsim provides steady state solutions and transition graphs for two different initial conditions: when CycD=0 and CycD=1. For the synchronous strategy, the transition graph can be visualized whereas for the asynchronous strategy, it is not easy to read or use; BoolNet constructs two graphical representations of the trajectories based on synchronous update strategy, for the case of CycD=0 (steady state) and CycD=1 (cycle); (3) MaBoSS estimates indecomposable stationary distributions for the case of CycD=0 (one fixed point, not shown) and CycD=1 (distribution of probabilities of different network states), and time-dependent activities of the cyclins showing damped oscillations. All results are coherent but are presented differently with a different focus for each tool. [file 1752-0509-6-116-S2.pdf]
